# Phenotypic and functional characterization of first-trimester human placental macrophages, Hofbauer cells

**DOI:** 10.1084/jem.20200891

**Published:** 2020-10-19

**Authors:** Jake R. Thomas, Anna Appios, Xiaohui Zhao, Roksana Dutkiewicz, Maria Donde, Colin Y.C. Lee, Praveena Naidu, Christopher Lee, Joana Cerveira, Bing Liu, Florent Ginhoux, Graham Burton, Russell S. Hamilton, Ashley Moffett, Andrew Sharkey, Naomi McGovern

**Affiliations:** 1 Department of Pathology, University of Cambridge, Cambridge, UK; 2 Centre for Trophoblast Research, Departments of Physiology and Neuroscience, University of Cambridge, Cambridge, UK; 3 Singapore Immunology Network, Agency for Science, Technology and Research, Singapore, Singapore; 4 School of Biological Sciences, Nanyang Technological University, Singapore, Singapore; 5 Key Laboratory for Regenerative Medicine of Ministry of Education, Institute of Hematology, School of Medicine, Jinan University, Guangzhou, China; 6 State Key Laboratory of Proteomics, Academy of Military Medical Sciences, Academy of Military Sciences, Beijing, China; 7 State Key Laboratory of Experimental Hematology, Institute of Hematology and Blood Diseases Hospital, Chinese Academy of Medical Sciences, Tianjin, China; 8 Shanghai Institute of Immunology, Shanghai JiaoTong University School of Medicine, Shanghai, China; 9 Translational Immunology Institute, SingHealth Duke-NUS Academic Medical Centre, Singapore, Singapore; 10 Department of Genetics, University of Cambridge, Cambridge, UK

## Abstract

Hofbauer cells (HBCs) are a population of macrophages found in high abundance within the stroma of the first-trimester human placenta. HBCs are the only fetal immune cell population within the stroma of healthy placenta. However, the functional properties of these cells are poorly described. Aligning with their predicted origin via primitive hematopoiesis, we find that HBCs are transcriptionally similar to yolk sac macrophages. Phenotypically, HBCs can be identified as HLA-DR^−^FOLR2^+^ macrophages. We identify a number of factors that HBCs secrete (including OPN and MMP-9) that could affect placental angiogenesis and remodeling. We determine that HBCs have the capacity to play a defensive role, where they are responsive to Toll-like receptor stimulation and are microbicidal. Finally, we also identify a population of placenta-associated maternal macrophages (PAMM1a) that adhere to the placental surface and express factors, such as fibronectin, that may aid in repair.

## Teaser

Hofbauer cells are primitive placental macrophages with a unique phenotype and role in fetal defense. Using transcriptomic and proteomic data, Thomas et al. analyze human first-trimester placental macrophages and delineate markers that identify them. They also reveal that Hofbauer cells have microbicidal capacity, providing the fetus with an additional layer of protection from certain microbes.

## Graphical Abstract

**Figure:**
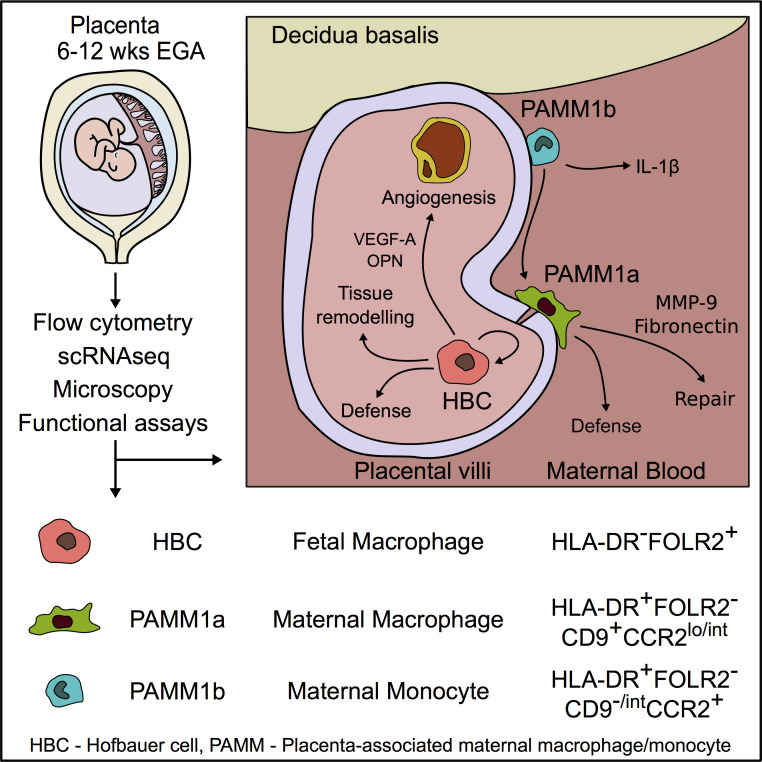

## Introduction

Macrophages are found within all human tissues, where, within the adult, they mediate tissue homeostasis, development, repair, and immunity. During embryonic development, the first macrophages to seed all tissues are derived through a process called primitive hematopoiesis. These macrophages, commonly termed “primitive” macrophages, are distinct from those generated through definitive hematopoiesis, as there is no monocyte intermediate (Ginhoux et al., 2010; Gomez Perdiguero et al., 2015). Although in some species, such as the mouse, primitive hematopoiesis is thought to only occur within the yolk sac (YS), during human embryonic development, primitive hematopoiesis also takes place in the placenta (Van Handel et al., 2010).

**Figure 1.**
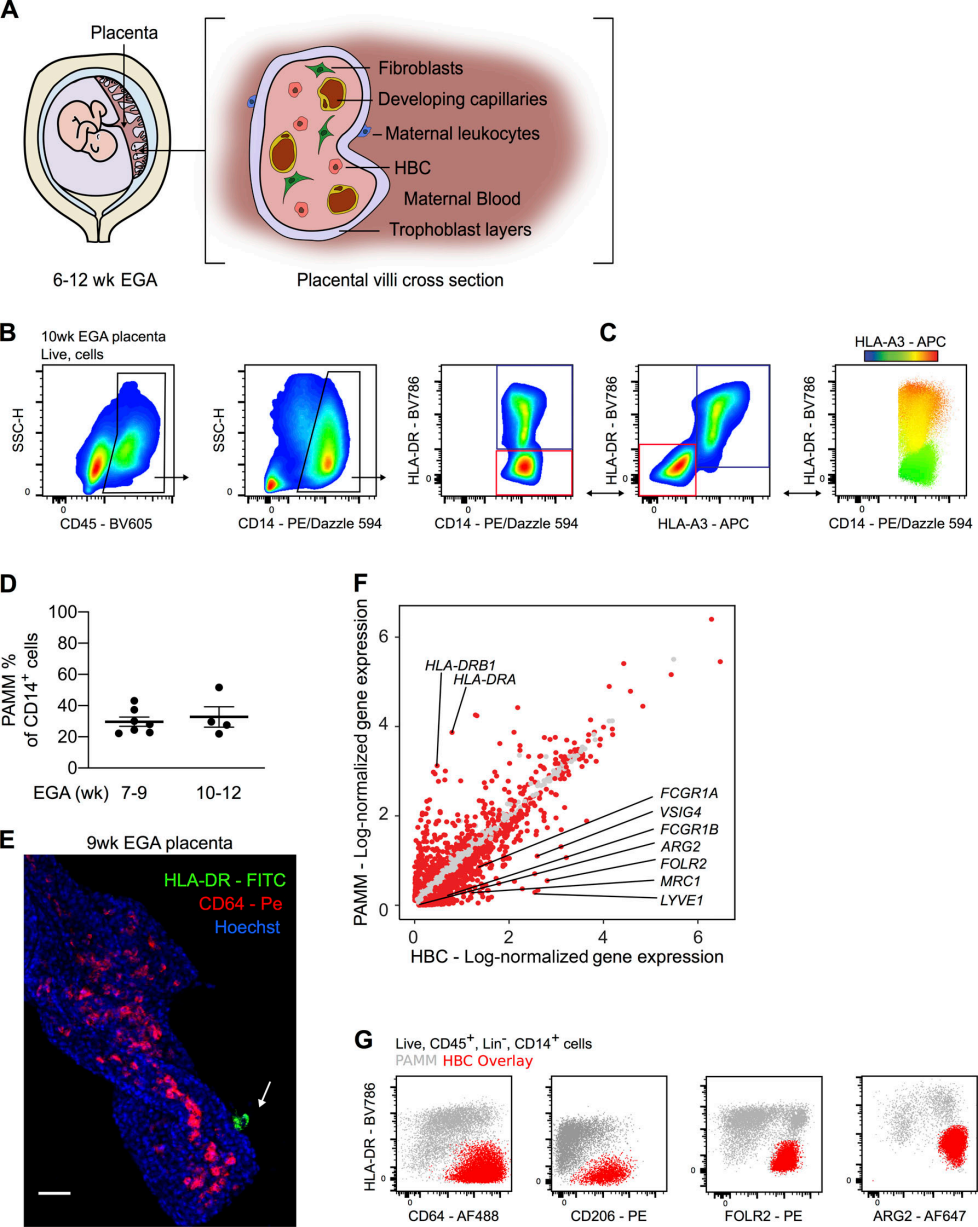
**Anti-HLA antibodies allow for the specific identification of HBCs by flow cytometry.** **(A)** Schematic drawing of the human placenta and a villous cross section. **(B)** Representative flow cytometric gating strategy identifying two placental macrophage populations based on HLA-DR expression. Blue gate, HLA-DR^+^ macrophages. Red gate, HLA-DR^−^ macrophages. **(C)** Differential expression of HLA-A3 within the CD14^+^ macrophage gate, shown by biaxial plot and heatmap overlay. Maternal macrophages are indicated by the blue gate (HLA-DR^+^HLA-A3^+^), and fetal macrophages are indicated by the red gate (HLA-DR^−^HLA-A3^−^). Bidirectional arrows depict equivalent cells. **(D)** Quantification of the abundance of PAMM within CD14^+^ placental cell suspensions across the indicated EGA. Each data point indicates a separate donor (*n* = 11). **(E)** Whole-mount immunofluorescence of a placental villus, where HBCs stained with CD64 (red) are within villous stroma and PAMMs stained with HLA-DR (green, white arrow) are on the syncytial layer. Cell nuclei are stained with Hoechst (blue). Scale bar, 50 µm. Representative image of *n* = 3 experiments. **(F)** Scatterplot showing log-normalized gene expression of HBC (x axis) and PAMM (y axis) clusters derived from scRNA-seq data analysis. Red dots represent genes that are differentially expressed with an adjusted P value < 0.01 (Wilcoxon rank sum test). **(G)** Flow cytometric analysis of expression of indicated markers by HBCs (identified with anti-HLA antibodies in red overlay) and PAMMs (gray). Representative plots of *n* = 3 experiments. Data are represented as mean ± SEM (D). SSC-H, side scatter height.

The placenta is a major organ that regulates the health of both the mother and developing fetus during pregnancy. The human placenta develops from the trophoectoderm, the outer layer of the preimplantation blastocyst, which forms at ∼5 d postfertilization (dpf; Turco and Moffett, 2019). As the placenta develops, highly branched villous tree-like structures form, which contain fibroblasts, immature capillaries, and macrophages, termed Hofbauer cells (HBCs; Fig. 1 A). The mesenchymal core is surrounded by a bilayer of specialized placental epithelial cells called trophoblasts. The outermost syncytiotrophoblast (SCT) layer, in contact with maternal blood, is formed by fusion of underlying cytotrophoblast cells (Turco and Moffett, 2019). HBCs have been identified within the placenta around day 18 after conception (Castellucci et al., 1987; Boyd and Hamilton, 1970), before the placenta is connected to the embryonic circulation (Van Handel et al., 2010).

A number of recent studies have profiled the gene expression of human embryonic macrophage populations (Stewart et al., 2019; Vento-Tormo et al., 2018). However, studies demonstrating their functional properties remain limited. Our previous work demonstrating that second-trimester fetal dendritic cells are functionally active and responsive to TLR stimulation (McGovern et al., 2017) led us to query if primitive macrophages have similar capabilities. In particular, we were interested in determining if HBCs demonstrate microbicidal capacity, as they are the only fetal immune cells found within the stroma of the human placenta, the crucial tissue barrier site between maternal tissues and the fetus.

In this study, we sought to develop a technique that would allow us to characterize the properties of HBCs isolated from first-trimester human placentas. Using a novel flow cytometric gating strategy, we find that commonly used protocols for the isolation of HBCs from first-trimester placentas yield a heterogenous population of macrophages that also consist of placenta-associated maternal monocyte/macrophage (PAMM) subsets. We demonstrate that HBCs have a unique phenotype specific to the placental niche; they do not express HLA-DR and highly express folate receptor 2 (FOLR2). We identify a range of factors that HBCs secrete that possibly affect placental angiogenesis and remodeling, including IL-8, osteopontin (OPN), and matrix metalloproteinase 9 (MMP-9). We show that HBC are responsive to TLR stimulation and do have microbicidal capacity and can thereby play a defensive role for the fetus. Finally, we identify a novel population of placenta-associated maternal macrophages (PAMM1a) that could function in tissue repair. Our findings provide novel insights into the properties of human primitive macrophages, and the roles of HBC in placental homeostasis.

## Results

### Identification of HBCs using anti-HLA antibodies

**Figure S1.**
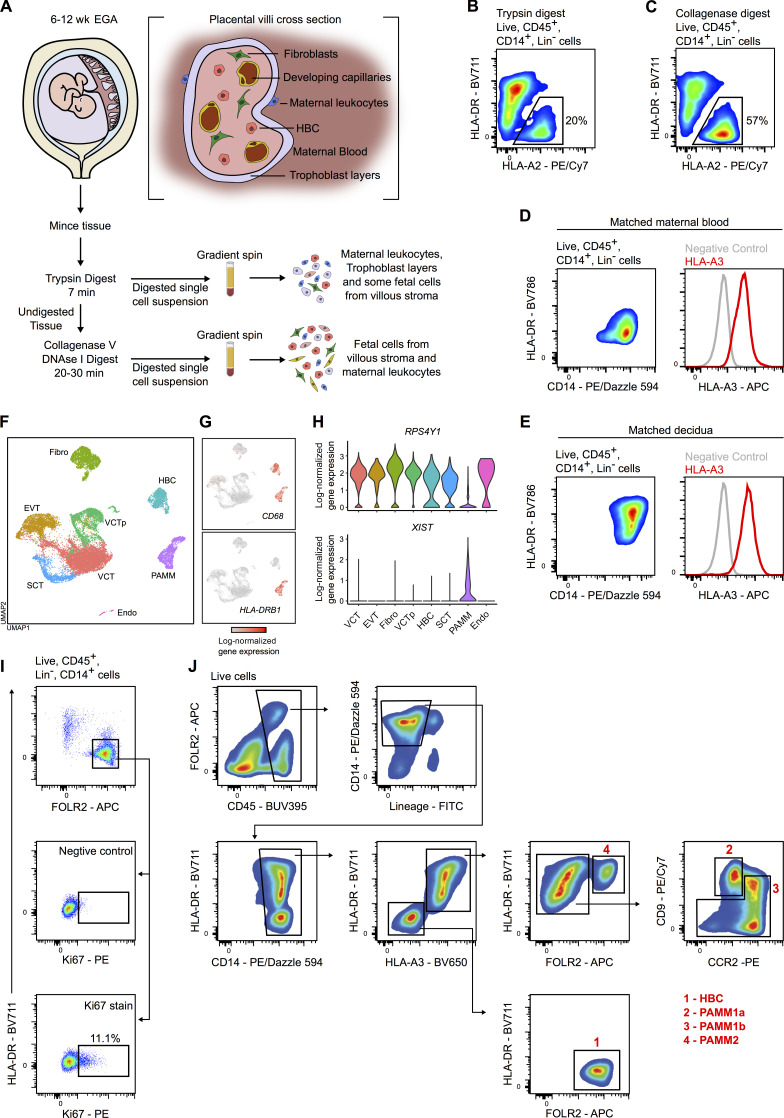
**Isolation and characterization of placental macrophage populations.** **(A)** Schematic representation of a digestion protocol used to isolate placental cells (Tang et al., 2011). **(B and C)** Flow cytometric analysis of fetal myeloid cells (HLA-A2^+^) from the same sample digested with either trypsin alone (B) or trypsin and collagenase (C). HBCs (black gate) and PAMMs are identified in both steps of the digestion process. **(D and E)** Flow cytometric analysis of maternal peripheral blood monocytes (D) and decidual CD14^+^ cells (E), matched with the placental sample shown in Fig. 1, B and C. **(F)** UMAP visualization of 22,618 placental single-cell transcriptomes (Vento-Tormo et al., 2018). Endo, endothelial cells; EVT, extravillous trophoblast; Fibro, fibroblasts; VCT, villous cytotrophoblast; VCTp, proliferating villous cytotrophoblast. **(G)** UMAP visualization with overlays of *CD68* and *HLA-DRB1* log-normalized gene expression. **(H)** Violin plots showing log-normalized gene expression of *RSP4Y1* and *XIST* for one male fetal donor from the scRNA-seq dataset. **(I)** Flow cytometric plots showing the gating strategy and representative Ki67 staining for HBCs (*n* = 8). **(J)** Representative flow cytometric plots for gating strategy used to isolate HBC and PAMM populations for phenotypic, morphological, and functional analysis. For the donor shown, maternal and fetal cells are HLA-A3^+^ and HLA-A3^−^, respectively.

Previous reports phenotyping HBCs isolated from the placenta have yielded conflicting results (Sutton et al., 1983; Böckle et al., 2008; Bulmer et al., 1988; Goldstein et al., 1988; Reyes and Golos, 2018). We first sought to determine the true identity of first-trimester HBC using multiparameter flow cytometry. Using a commonly used protocol to isolate HBCs (Tang et al., 2011; Fig. S1 A), we obtain a CD45^+^CD14^+^ macrophage population that is heterogeneous for HLA-DR expression (Fig. 1 B). We sought to determine if the observed heterogeneity is due to maternal monocyte/macrophage populations contaminating the HBC placental isolates.

Fetal cells express both maternally and paternally derived genes. To determine if maternal cells contaminate the HBC preparations and contribute to the heterogeneity in observed HLA-DR expression, we added antibodies to common HLA allotypes (HLA-A3, HLA-B7, and HLA-A2) to our flow cytometry panel. The specificity of these antibodies was previously verified by quantitative PCR (qPCR) on DNA from blood samples of HLA-typed donors. We chose to use anti-HLA antibodies instead of sex chromatin staining (to identify male fetal cells) so that we could develop a flow cytometry panel to allow isolation of live cells for functional assays. By using anti-HLA antibodies and analyzing matched maternal blood or decidual cells (Fig. S1, D and E), we consistently observe that the variable population of HLA-DR^+^ cells in first-trimester placental digests are maternal in origin (Fig. 1 C and Fig. S1, A–C). We termed the maternal cells obtained in placenta digests PAMMs.

We had expected that maternal contamination of placental macrophage cell isolates would only become significant from the 10th week of gestation, the time when maternal blood flow to the intervillous space is fully established (Burton et al., 2009b). However, application of our new gating strategy to placental digests of 7–9 wk estimated gestational age (EGA) and 10–12 wk EGA demonstrated that maternal cells make a significant contribution to the CD14^+^ macrophage populations isolated from the placenta as early as the 7th week of gestation, comprising 20–40% of CD14^+^ cells (Fig. 1 D). Whole-mount immunofluorescence microscopy revealed that while HLA-DR^+^ cells do adhere to the SCT layer of the placental villi, HLA-DR^+^ cells are not present within the stromal core from the 7th to 10th week of gestation (Fig. 1 E). These findings are in line with recent single-cell RNA sequencing (scRNA-seq) studies of placental cell isolates (Tsang et al., 2017; Vento-Tormo et al., 2018) where maternal cells were also observed. Our data demonstrate that maternal cell contamination is higher than has previously been appreciated within first-trimester placental cell suspensions. These findings will have a significant impact on in vitro studies that aim to determine the specific functional properties of HBC.

### Identification of specific markers for HBCs

A limitation of using anti-HLA antibodies is that they can only distinguish maternal and fetal cells where there is a maternal/fetal HLA mismatch for these specific allotypes. We therefore sought to identify markers that would allow us to confidently distinguish maternal from fetal cells independently of HLA antibodies. To do this, we performed transcriptomic analysis of first-trimester placental cells using a publicly available scRNA-seq dataset (Vento-Tormo et al., 2018). Clustering and Uniform Manifold Approximation and Projection (UMAP) visualization of 22,618 placental single cells identifies two distinct macrophage populations, as indicated by *CD68* expression (Fig. S1, F and G). Consistent with our flow cytometry analysis, PAMM highly express *HLA-DRB1* while HBCs do not express *HLA-DRB1* (Fig. S1 G). Expression of male (*RPS4Y1*)- and female (*XIST*)-specific genes in placental cells from male fetal donors confirms the fetal and maternal origin of HBC and PAMM clusters (Fig. S1 H).

There are 962 significantly differentially expressed genes (DEGs; adjusted P value < 0.01) between PAMM and HBC clusters. *FCGR1A*, *FCGR1B*, *VSIG4*, *MRC1*, *FOLR2*, and *LYVE-1* are up-regulated within HBCs. In contrast to HBC, PAMMs highly express *HLA-DRB1* and *HLA-DRA* (Fig. 1 F). We tested a number of additional HBC-specific markers that were identified by analysis of the sequencing data, including FOLR2, CD64, and CD206. We also analyzed the expression of arginase 2, which has previously been shown to be expressed by fetal immune cells (McGovern et al., 2017). Samples from donors where the anti-HLA antibodies distinguished maternal from fetal cells were used. Flow cytometric analysis demonstrates that these markers are expressed by HBCs at the protein level (Fig. 1 G). Any of these four markers, in combination with HLA-DR, allows us to confidently distinguish HBCs from PAMMs within first-trimester samples. The combination of FOLR2 and HLA-DR provides the clearest separation for the isolation of HBCs.

### HBCs are transcriptionally similar to primitive macrophages and proliferate in situ

**Figure 2.**
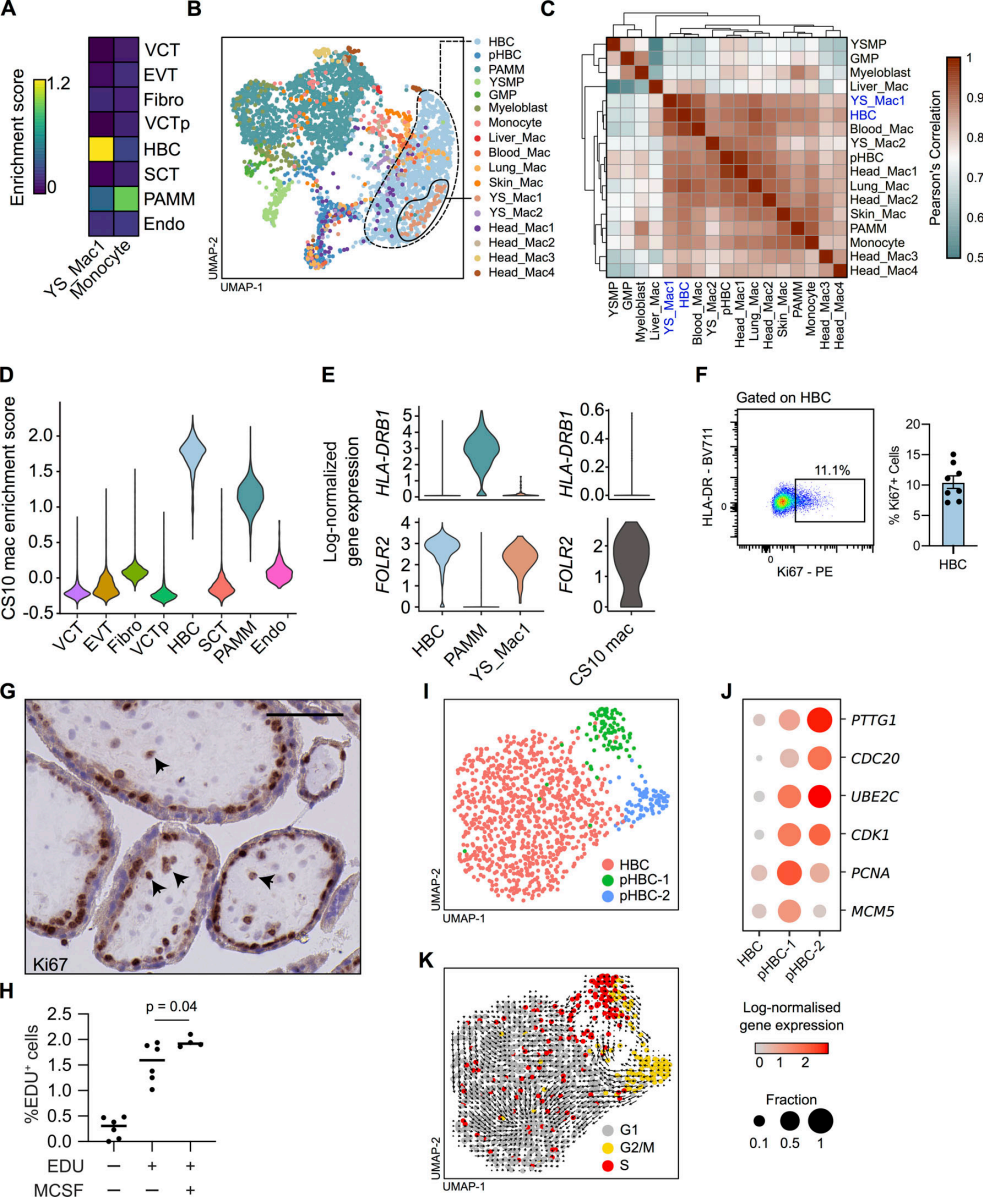
**First-trimester HBCs are transcriptionally similar to primitive macrophages and proliferate in situ.** **(A)** Heatmap of placental scRNA-seq cluster mean enrichment scores for extraembryonic YS macrophage and embryonic monocyte gene signatures (Bian et al., 2020). Endo, endothelial cells; EVT, extravillous trophoblast; Fibro, fibroblasts; VCT, villous cytotrophoblast; VCTp, proliferating villous cytotrophoblast. **(B)** UMAP visualization of 3,846 single-cell transcriptomes from first-trimester placenta and embryonic myeloid cells (Bian et al., 2020). GMP, granulocyte–monocyte progenitors; pHBC, proliferating HBCs; YS_Mac, YS macrophage; YSMP, YS-derived myeloid-biased progenitors. **(C)** Heatmap depicting transcriptomic similarity between annotated clusters. Clusters are ordered according to hierarchical clustering. HBCs and YS_Mac1s are highlighted in blue. **(D)** Violin plot of placental scRNA-seq cluster enrichment scores for primitive macrophages from a CS10 embryo (Zeng et al., 2019). **(E)** Violin plots of *HLA-DRB1* and *FOLR2* log-normalized gene expression in HBCs, PAMMs, YS_Mac1s, and CS10 macrophages. **(F)** Representative flow cytometric plot and quantification of Ki67 expression by HBCs (*n* = 8). **(G)** Representative immunohistochemistry analysis of Ki67 expression in placental tissue sections. Arrowheads indicate Ki67^+^ cells. Scale bar, 100 µm. **(H)** Incorporation of EdU into FACS-isolated HBCs after 18 h culture, with and without the addition of M-CSF (*n* ≥ 4); P value was calculated by one-way ANOVA. **(I)** UMAP visualization of 1,091 HBC single-cell transcriptomes identifying two proliferating HBC populations. **(J)** Dotplot heatmap of log-normalized gene expression of genes associated with stages of the cell cycle in HBC clusters. Dot size represents fraction of cells with nonzero expression. **(K)** UMAP visualization of HBCs with cells colored by predicted cell cycle state, as determined by cell cycle scoring, with RNA velocity vector field projection calculated from all genes in all cells (arrows) overlain. Data are represented as mean ± SEM (F) or mean alone (H).

HBCs are predicted to be primitive macrophages derived directly from progenitors independent of monocytes. A recent study characterizing the transcriptional landscape of human macrophage development identified a population of true primitive YS macrophages (YS_Mac1) from a Carnegie stage 11 embryo (∼4 wk after conception; Bian et al., 2020). Consistent with their predicted primitive origins, HBCs are enriched for a gene signature derived from YS_Mac1, but not embryonic monocytes (Fig. 2 A and Data S1). Integration of first-trimester placental (Vento-Tormo et al., 2018) and early human fetal myeloid scRNA-seq (Bian et al., 2020) datasets reveals a high degree of transcriptional similarity between HBCs and primitive YS_Mac1 (Fig. 2, B and C). PAMMs, however, display transcriptional similarity to embryonic monocytes, reflecting their monocytic origins (Fig. 2, A and C).

HBCs are also highly enriched for a gene signature from YS-derived embryonic macrophages from a Carnegie stage 10 embryo (∼4 wk after conception; CS10 mac) from an additional dataset (Zeng et al., 2019; Fig. 2 D and Data S1). PAMMs display intermediate levels of enrichment for the CS10 mac gene signature. This is likely due to conserved myeloid genes not specific to primitive macrophages within the gene signature, as it was generated via comparison between CS10 macs and nonimmune cells in that dataset (Materials and methods). Analysis of individual genes reveals further similarity among HBCs, YS_Mac1, and CS10 mac on the basis of *HLA-DRB1* and *FOLR2* expression (Fig. 2 E).

Due to the transcriptional similarity between HBCs and primitive macrophages, we hypothesized that HBCs would be maintained in the tissue via local proliferation. We find that ∼11% of freshly isolated HBCs express Ki67 by flow cytometry (Fig. 2 F and Fig. S1 I) and identify Ki67^+^ cells within the stroma of placental villi by immunohistochemistry (Fig. 2 G). Furthermore, during overnight culture, ∼1.5% of FACS-isolated HBCs incorporate 5-ethynyl-2′-deoxyuridine (EdU), and incorporation is slightly elevated by the addition of M-CSF to the cultures (Fig. 2 H). Directed analysis of the HBC cluster within the placental scRNA-seq dataset identifies two proliferating populations, pHBC-1 and pHBC-2 (Fig. 2 I). These clusters express genes associated with distinct stages of the cell cycle (*PTTG1*, *CDC20*, *UBE2C*, *CDK1*, *PCNA*, and *MCM5*; Fig. 2 J), and cell cycle scoring assigns pHBC-1 and pHBC-2 to the S and G2/M phases of the cell cycle, respectively (Fig. 2 K). RNA velocity vectors, derived by calculating the ratio between spliced and unspliced reads of each gene within each cell (La Manno et al., 2018), demonstrate a clear path of HBCs through the cell cycle (Fig. 2 K). No subpopulations are observed within nonproliferating HBCs, allowing us to isolate them as a single population for functional assays.

Together, these data show that HBCs are transcriptionally similar to macrophage populations generated through primitive hematopoiesis and are a homogenous population, proliferating within placental villi, suggesting that they arise without a monocyte intermediate.

### PAMMs are heterogeneous

**Figure 3.**
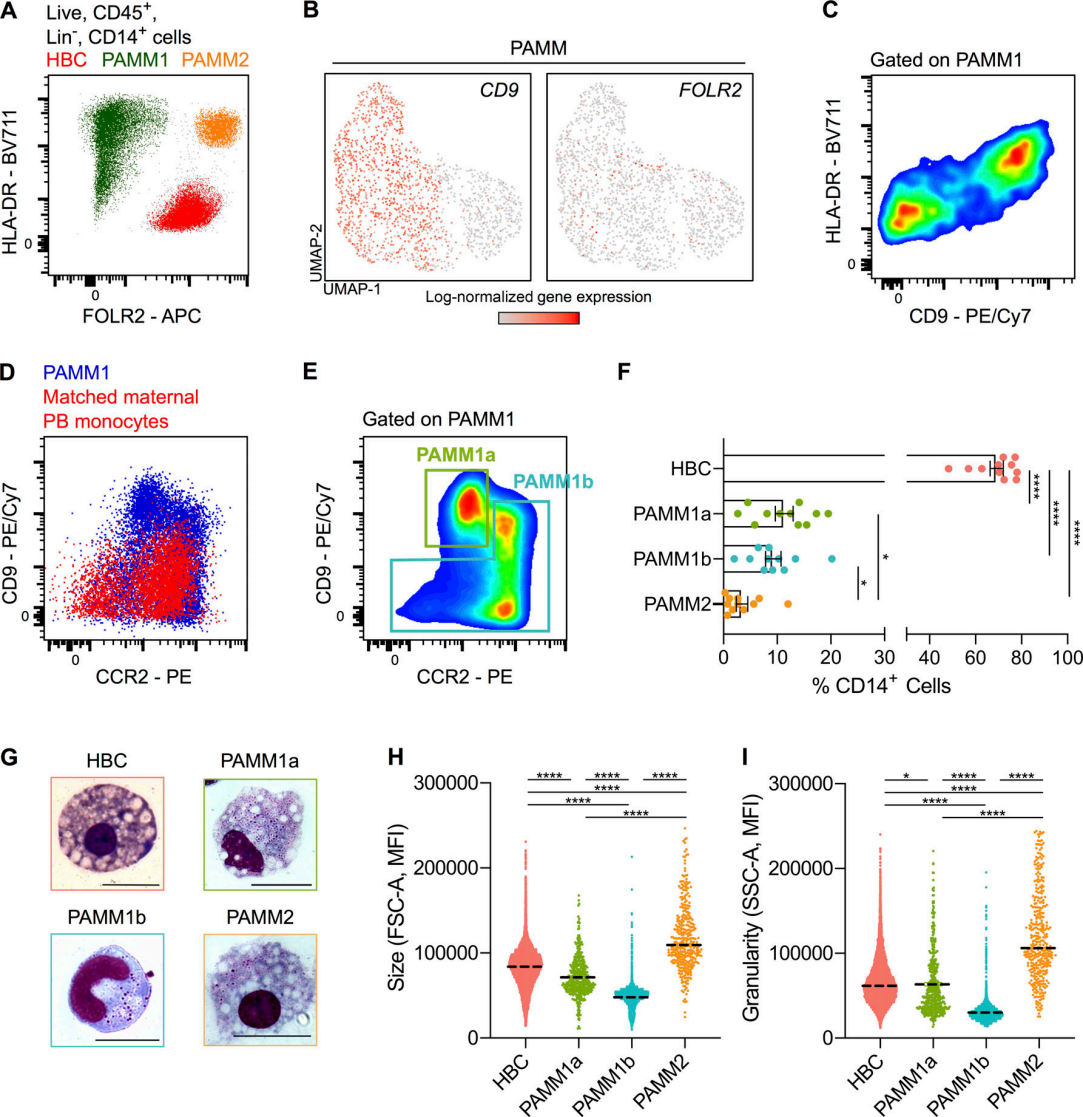
**PAMMs are a heterogeneous population comprised of three subsets based on their expression of FOLR2, CD9 and CCR2 expression. (A)** Expression of FOLR2 and HLA-DR by flow cytometry reveals three major populations of placental macrophages: HBC (red), PAMM1 (green), and PAMM2 (orange). **(B)** UMAP visualization of 1,687 PAMM single-cell transcriptomes from first-trimester placenta, with overlays of *CD9* and *FOLR2* log-normalized gene expression. **(C)** Heterogeneous expression of CD9 within PAMM1 by flow cytometry. **(D)** Overlay flow cytometric plots of PAMM1 (blue) and peripheral blood (PB) monocytes from matched maternal blood (red) of CD9 and CCR2 expression. **(E)** Flow cytometric plot of CD9 and CCR2 expression within PAMM1s, showing representative gates for the identification of PAMM1a and PAMM1b. **(F)** Enumeration of HBC and PAMM populations as a percentage of total CD14^+^ cells from placental cell suspensions (*n* = 11). P values were calculated by one-way ANOVA with Tukey’s multiple-comparisons test. **(G)** Representative Giemsa-Wright–stained cytospins of HBC and PAMM subsets isolated by FACS. Scale bars, 20 µm. **(H)** Forward scatter (FSC-A) and (I) side scatter (SSC-A) mean fluorescence intensity (MFI) of HBC and PAMM subsets. P values were calculated by one-way ANOVA with Tukey’s multiple-comparisons test. Data are represented as mean ± SEM (F) or mean alone (H and I). *, P ≤ 0.05; ****, P ≤ 0.0001.

Our flow cytometric analysis clearly shows that PAMMs consist of two major populations: HLA-DR^hi/lo^FOLR2^−^ cells (PAMM1s) and HLA-DR^hi^FOLR2^hi^ cells (PAMM2s; Fig. 3 A). Subsequently, directed reanalysis of PAMMs within the scRNA-seq dataset reveals further heterogeneity on the basis of *CD9*, but not *FOLR2*, expression (Fig. 3 B). Adding CD9 to our flow cytometry panel, the HLA-DR^hi/lo^FOLR2^−^ (PAMM1) cells are split into two populations (Fig. 3 C). To determine if either of these populations are circulating maternal monocytes, we added the monocyte maker CCR2 to the panel and performed flow cytometry on matched maternal blood. FOLR2^−^CD9^hi^CCR2^lo/int^ cells are not present in matched maternal blood, indicating that they are macrophages with a phenotype specific to the placental niche. The remaining PAMM1 cells do, however, share a similar phenotype with maternal peripheral blood monocytes (Fig. 3 D). Therefore, we subdivided PAMM1s into two populations: PAMM1a (FOLR2^−^CD9^hi^CCR2^lo/int^; macrophages) and PAMM1b (FOLR2^−^CD9^-/int^CCR2^+^; monocytes; Fig. 3 E and Fig. S1 J).

**Figure S2.**
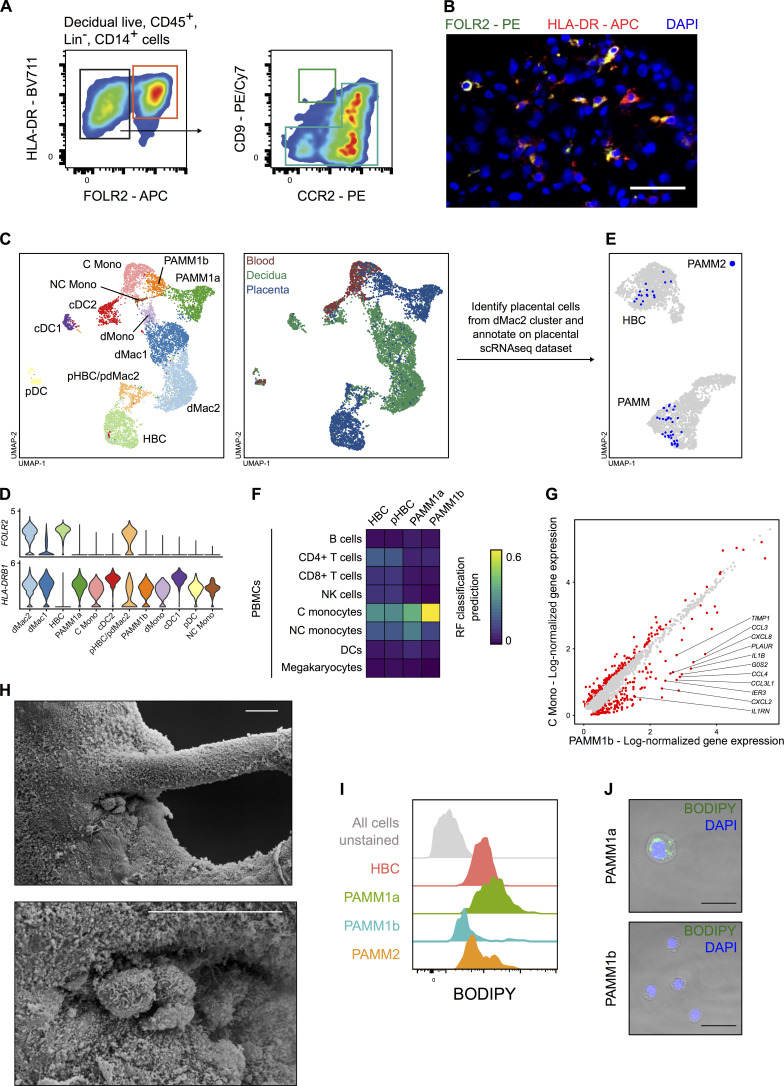
**Identification of PAMM populations.** **(A)** Flow cytometric analysis of decidual CD14^+^ cells. Cells with a phenotype consistent with PAMM2 (FOLR2^+^ HLA-DR^+^; red gate) and PAMM1b (blue gate) were readily identified. Cells with a phenotype consistent with PAMM1a were low in abundance (green gate). Representative flow cytometric plots from *n* = 3 experiments. **(B)** Identification of HLA-DR^+^ (red) FOLR2^+^ (green) macrophages in the decidua by fluorescence microscopy. Cell nuclei are stained with DAPI (blue). Scale bar, 50 µm. Representative image of *n* = 2 experiments. **(C)** UMAP visualization of 9,474 myeloid cells from placenta, decidua, and maternal blood (Vento-Tormo et al., 2018). Cells are colored and labeled by cluster identity (left panel) and tissue of origin (right panel). cDC1, conventional type 1 dendritic cells; cDC2, conventional type 2 dendritic cells; C mono, classical monocytes; dMac1, decidual macrophages 1; dMac2, decidual macrophages 2; dMono, decidual monocytes; NC Mono, nonclassical monocytes; pDC, plasmacytoid dendritic cells; pHBC/pdMac2, proliferating HBCs and dMac2s. **(D)** Violin plots showing log-normalized gene expression of *FOLR2* and *HLA-DRB1* in placental, decidual, and maternal blood myeloid cells. **(E)** Annotation of PAMM2 (placental cells within dMac2 cluster; blue) onto original UMAP embedding of HBCs and PAMMs from the placental scRNA-seq dataset (Fig. S1 F). **(F)** Heatmap of transcriptional similarity between placental macrophage/monocyte cell clusters and indicated PBMC populations, as determined using a random forest (RF) classification prediction. DC, dendritic cell; NK, natural killer; p-HBC, proliferating HBC. **(G)** Scatterplot showing log-normalized gene expression of PAMM1b (x axis) and maternal blood classical monocyte (y axis) clusters. Red dots represent genes that are differentially expressed with an adjusted P value < 0.01 (Wilcoxon rank sum test). **(H)** Scanning electron micrographs of PAMM1a on the surface of a first-trimester placenta, adhering to a site of damage on a branching villus. Scale bars, 20 µm. **(I)** Representative flow cytometric histograms of BODIPY staining within HBC and PAMM subsets compared with unstained cells (gray). **(J)** Images of BODIPY staining of FACS-isolated PAMM1a and PAMM1b. Scale bars, 20 µm. Representative images of *n* = 2 experiments.

HLA-DR^hi^FOLR2^hi^ cells (PAMM2s) are rare in placental samples (∼3% of placental CD14^+^ cells; Fig. 3 F). Decidual macrophages also express FOLR2 and HLA-DR (Fig. S2, A and B), and it is likely that PAMM2s are maternal decidual macrophages that will contaminate placental samples. Although PAMM2s do not form a distinct cluster in the placental scRNA-seq dataset, combined analysis of placental, decidual, and maternal blood scRNA-seq datasets (Fig. S2 C) reveals that HLA-DR^+^ FOLR2^+^ decidual macrophages (dMac2; Fig. S2 D) are found in low numbers in the placental digests (Fig. S2 E; Vento-Tormo et al., 2018). Cytospins and analysis of cell granularity and size by flow cytometry shows that HBCs, PAMM1a, and PAMM2 are large, granular cells with morphologies typical of macrophages (large vacuoles and pseudopods; Fig. 3, G–I). PAMM1b’s are comparatively smaller in size, and their morphology is typical of blood monocytes (Fig. 3, G–I). In line with their phenotypic and morphological properties, we find that PAMM1b’s are transcriptionally similar to adult circulating classical monocytes (Fig. S2 F). However, PAMM1b’s display increased expression of 150 genes, including chemokines, in comparison to maternal blood classical monocytes (Fig. S2 G). PAMM1a’s are not present within the decidua (Fig. S2 A), indicating their phenotype probably reflects adherence to the SCT. Of the three PAMM populations identified, PAMM1a’s are the most abundant, representing ∼11% of the CD14^+^ cells in placental isolates (Fig. 3 F).

In conclusion, PAMMs can be subdivided into different populations. PAMM1b’s are monocytes, PAMM1a’s are macrophages that are specific to the placental surface, and PAMM2s are contaminating decidual macrophages.

### PAMM1a’s adhere to sites of injury on the placental surface and secrete factors involved in tissue repair

**Figure 4.**
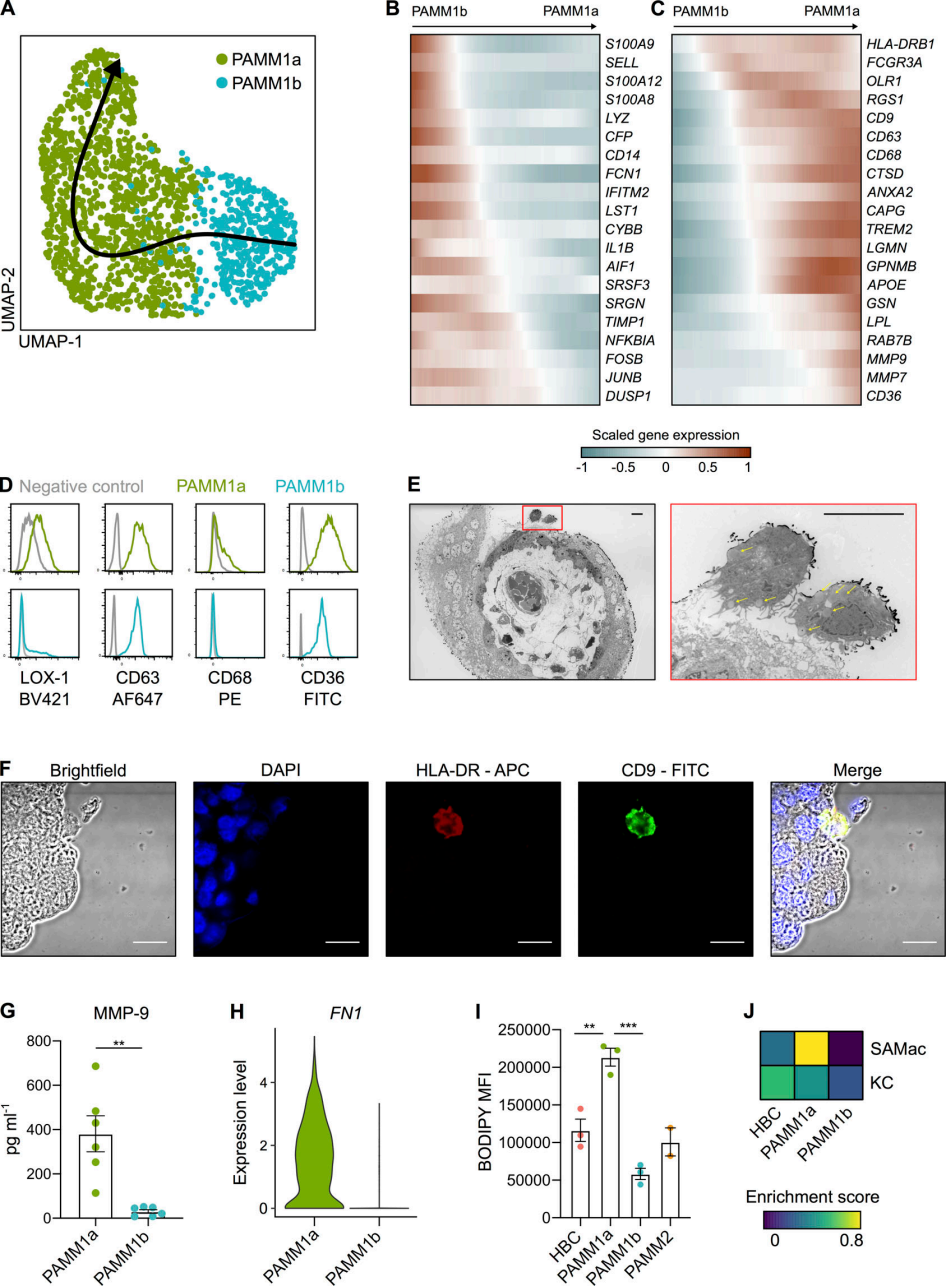
**PAMM1 undergo a monocyte-to-macrophage transition and adopt a tissue-repair phenotype on the placental surface.** **(A)** UMAP visualization of 1,687 PAMM single-cell transcriptomes with Slingshot trajectory overlain. **(B and C)** Heatmaps of smoothed scaled gene expression of selected genes that are down-regulated (B) and up-regulated (C) during PAMM1b to PAMM1a differentiation, ordered according to Slingshot trajectory. **(D)** Relative surface expression of markers identified in C in PAMM1a (green) and PAMM1b (cyan), compared with fluorescence minus one (FMO) control (gray), measured by flow cytometry. Representative plots of *n* = 3. **(E)** Transmission electron microscopy of first-trimester placental villous cross section. PAMM1a can be observed on the placental surface, localized to sites of damage to the syncytial layer (red inset). PAMM1a’s are loaded with lipid droplets (arrows). Scale bars, 20 µm. **(F)** Identification of CD9^+^ (green) HLA-DR^+^ (red) PAMM1a cells on the surface of a 9-wk EGA placental sample by fluorescence microscopy. Representative image of *n* = 3 experiments. Cell nuclei are stained with DAPI (blue). Scale bars, 20 µm. **(G)** Secretion of MMP-9 by FACS-isolated PAMM1a and PAMM1b after overnight culture (*n* = 6). P value calculated by unpaired *t* test. **(H)** Log-normalized gene expression of fibronectin (*FN1*) in PAMM1a and PAMM1b clusters, as determined from scRNA-seq data. **(I)** Analysis of intracellular neutral lipid content by flow cytometry following staining with BODIPY; mean fluorescence intensity (MFI) of HBC and PAMM subsets is shown. P values calculated by one-way ANOVA with Tukey’s multiple-comparisons test. **(J)** Heatmap of placental macrophage mean enrichment scores for KC and SAMac gene signatures (Ramachandran et al., 2019). Data are represented as mean ± SEM. **, P ≤ 0.01; ***, P ≤ 0.001.

We next sought to determine the potential role of PAMM1a in healthy pregnancy. To investigate what changes in gene expression occur during the transition from PAMM1b (monocytes) to PAMM1a (macrophages), we performed Slingshot trajectory analysis (Street et al., 2018; Fig. 4 A). Genes associated with monocyte identity and function, including *S100A8*, *S100A9*, and *LYZ*, are down-regulated along the trajectory (Fig. 4 B). Up-regulated genes included macrophage markers *CD63*, *CD68*, *CD36*, and *GPNMB* and a subset of genes associated with tissue remodeling (including *LPL*, *MMP7*, and *MMP9*; Fig. 4 C). The elevated surface expression of LOX-1 (the receptor encoded by *OLR1*), CD63, CD68, and CD36 by PAMM1a are verified by flow cytometry (Fig. 4 D).

Breaks occur in the SCT in vivo in healthy pregnancies (Burton and Watson, 1997). Fibrin deposits together with macrophages are characteristically seen at the sites of syncytial damage (Pierleoni et al., 2003; Burton and Watson, 1997). We identify that PAMM1a adhered to sites of damage on the SCT by electron (Fig. 4 E and Fig. S2 H) and fluorescent microscopy (Fig. 4 F). PAMM1a’s secrete MMP-9 (detected by Luminex assay after FACS isolation and overnight culture; Fig. 4 G) and strongly express *FN1* (fibronectin; Fig. 4 H). Transmission electron microscopy reveals that PAMM1a’s are laden with lipid droplet–like structures (yellow arrows in Fig. 4 E). Staining with BODIPY, a dye that specifically labels neutral lipids, confirms that PAMM1a’s are highly loaded with lipid droplets (Fig. 4 I and Fig. S2, I and J). Lipid droplet formation in macrophages can be induced by the uptake of apoptotic cells (Ward et al., 2018; Ford et al., 2019; D’Avila et al., 2011), suggesting PAMM1a function in the clearance of cellular debris and repair of the SCT following damage. If this is the case, PAMM1a might display transcriptomic similarities to macrophages in damaged, fibrotic tissues. Indeed, we find that PAMM1a’s, but not HBCs, are strongly enriched for a gene signature from a population of scar-associated macrophages (SAMac) found in human cirrhotic livers (Ramachandran et al., 2019; Fig. 4 J and Data S1).

To summarize, we have identified PAMM1a’s on the SCT, and these cells are likely to function in essential repair of the placental barrier.

### HBCs produce factors that promote placental angiogenesis

**Figure 5.**
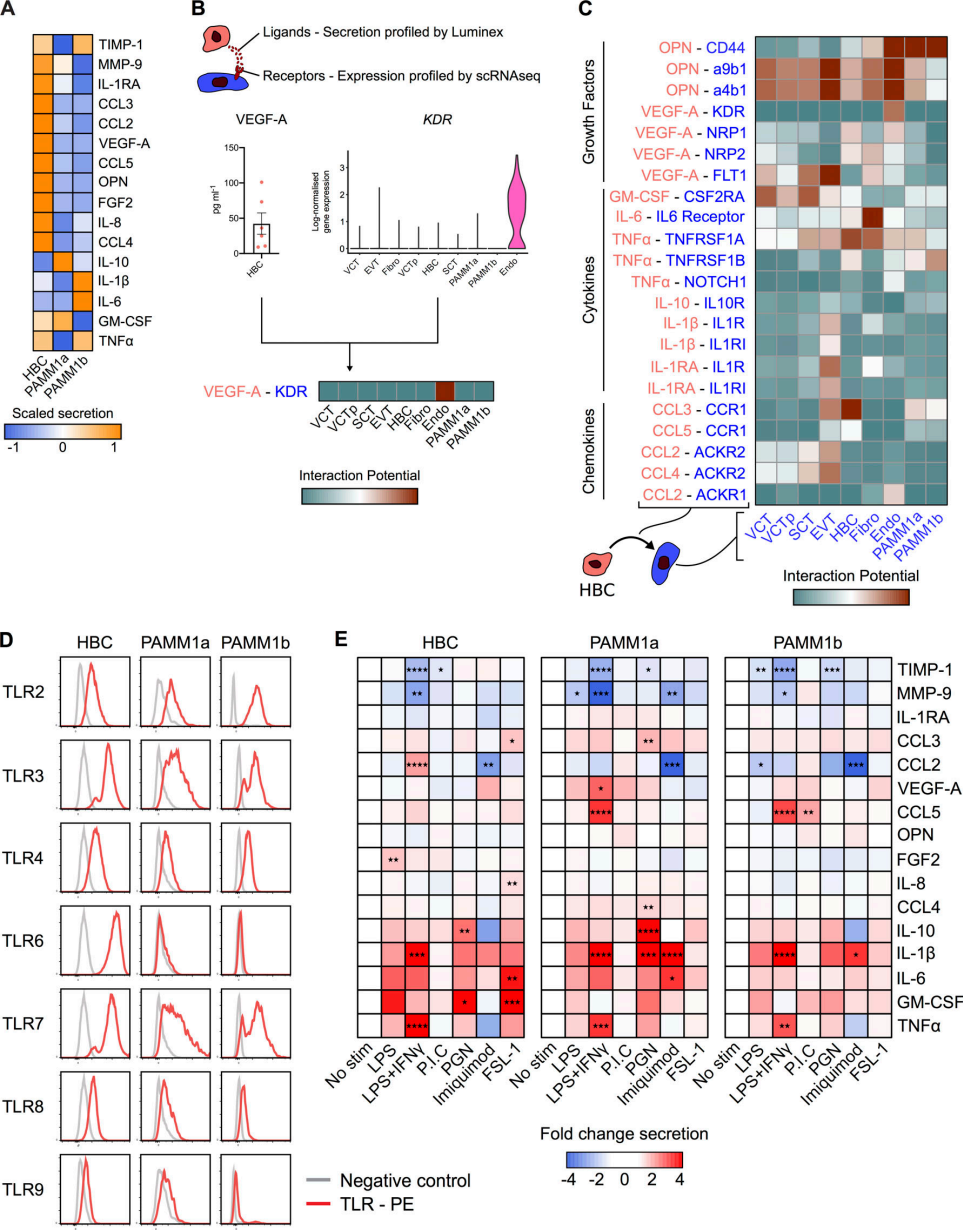
**HBC and PAMM subsets display distinct cytokine secretion profiles at the steady state and in response to TLR stimulation.** **(A)** Heatmap of average scaled cytokine, chemokine, and growth factor secretion from FACS-isolated HBCs, PAMM1a, and PAMM1b after overnight culture without stimulation (*n* = 6). **(B)** Schematic representation of inferred cell–cell interactions from Luminex and scRNA-seq data. Lower panel shows an example of predicted interactions between HBCs and other placental cells based on VEGF-A and kinase insert domain receptor (KDR). **(C)** Heatmap of predicted interactions between HBC (red) and other placental cell populations (blue). Interaction potentials were calculated from expression of ligands determined by protein secretion, and scRNA-seq expression of cognate receptors. **(D)** Relative flow cytometric expression of TLRs in HBCs, PAMM1a, and PAMM1b compared with FMO control (gray). Plots are representative of *n* = 3 experiments. **(E)** Heatmaps showing the fold change in cytokine secretion of FACS-isolated HBCs, PAMM1a, and PAMM1b cultured overnight with TLR stimulation relative to no stimulation (No stim; *n* = 6). PGN, peptidoglycan. P.I.C., polyinosinic-polycytidylic acid. P values were calculated by two-way ANOVA with Dunnett’s multiple-comparisons test. *, P ≤ 0.05; **, P ≤ 0.01; ***, P ≤ 0.001; ****, P ≤ 0.0001.

**Figure S3.**
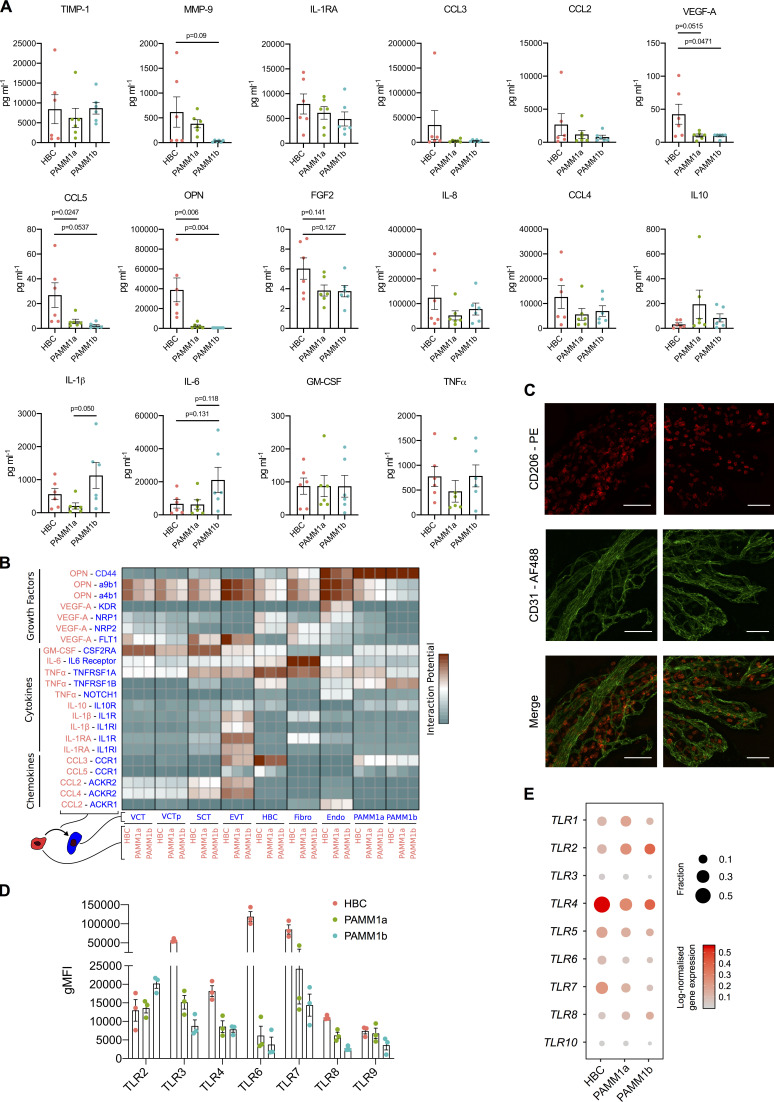
**HBC, PAMM1a, and PAMM1b secretome analysis in steady-state and TLR expression. (A)** Cytokine, chemokine, and growth factor secretion of FACS-isolated HBCs, PAMM1a, and PAMM1b after 18 h in culture without stimulation, profiled by Luminex (*n* = 6). P values were calculated by one-way ANOVA with Tukey’s multiple-comparisons test. Only significant P values and P values approaching significance are shown. **(B)** Heatmap of predicted interactions among HBCs, PAMM1a, and PAMM1b (red), and other placental cell populations (blue). Interaction potentials were calculated from expression of ligands determined by protein secretion and scRNA-seq expression of cognate receptors. Endo, endothelial cells; EVT, extravillous trophoblast; Fibro, fibroblasts; VCT, villous cytotrophoblast; VCTp, proliferating villous cytotrophoblast. **(C)** Whole-mount immunofluorescence of placental villi stained for CD206 (red) and CD31 (green). Images are from two independent donors, both at 9 wk EGA. Scale bar, 100 µm. **(D)** Quantification of expression of TLRs in HBCs, PAMM1a, and PAMM1b, as profiled by flow cytometry (*n* = 3). gMFI, geometric mean fluorescence intensity. **(E)** Dotplot heatmap of log-normalized gene expression of TLR genes in HBC and PAMM scRNA-seq clusters. Dot size represents fraction of cells with nonzero expression. TLR-9 was not detected in the analysis. Data are represented as mean ± SEM.

HBC, PAMM1a, and PAMM1b populations were isolated by FACS from placental digests and cultured overnight, and their secretion of cytokines and growth factors was determined by Luminex (PAMM2 cell yields were too low for functional assays; Fig. 5 A and Fig. S3 A). The secretion profile of PAMM1a and PAMM1b differs substantially from that of HBCs, reflecting their maternal origin. PAMM1b’s secrete increased amounts of the proinflammatory cytokines IL-1β and IL-6 in comparison with PAMM1a (Fig. 5 A), consistent with a monocyte to macrophage transition. In comparison with PAMM1a and PAMM1b, HBCs secrete both VEGF-A and low levels of FGF-2, growth factors involved in placental growth and angiogenesis (Burton et al., 2009a; Arany and Hill, 1998), as well as high levels of OPN, which has a role in implantation and placentation (Johnson et al., 2003). Surprisingly, HBCs also secrete factors that are typically associated with inflammation, such as IL-8, CCL-2, CCL-3, and CCL-4. However, these factors also have proangiogenic properties, a more likely role in the context of the placenta (Shi and Wei, 2016; Lien et al., 2018; Wu et al., 2008; Salcedo et al., 2000; Stamatovic et al., 2006). HBCs expressed tissue inhibitor of metalloproteinase 1 (TIMP-1) and MMP-9, both of which are involved in remodeling of placental vessels (Luizon et al., 2014; Plaks et al., 2013).

To determine which cells respond to factors secreted by HBCs, we generated a measure of the interaction potential between HBCs and other placental cells by combining Luminex protein secretion data with scRNA-seq gene expression data for cognate receptors (Fig. 5 B). Our analysis reveals predicted targets of HBC signaling (Fig. 5 C and Fig. S3 B). Endothelial cells are the main target of VEGF-A secretion, mediated by the expression of kinase insert domain receptor (*KDR*) and neuropilin 1 (*NRP1*). OPN is also predicted to signal to endothelial cells via *CD44* and integrin complexes, interactions that are known to promote angiogenesis (Dai et al., 2009; Poggio et al., 2011). HBC–endothelial cell interactions are also facilitated by their close proximity within placental villi (Fig. S3 C). Additionally, HBCs are predicted to signal to placental fibroblasts via IL-6 and to villous cytotrophoblasts via both OPN and GM-CSF.

In summary, we have identified factors that HBCs secrete that are likely to promote placental growth and homeostasis through interactions with endothelial cells, fibroblasts, and trophoblasts.

### HBCs are responsive to TLR stimulation

The placenta is a crucial barrier protecting the fetus from vertical infections, and HBCs are the only fetal myeloid cells in the first-trimester placenta. However, their role in defending the fetus from infection remains unclear. In addition, whether primitive macrophages have the capacity to detect and respond to microbial stimuli is unknown. We therefore next asked whether HBCs are responsive to TLR stimulation. TLRs drive specific immune responses through the recognition of distinct pathogen-associated molecular patterns derived from a range of microbes (Kawasaki and Kawai, 2014).

The TLR expression profile of HBCs analyzed by flow cytometry is distinct from PAMM populations (Fig. 5 D and Fig. S3 D); while HBCs express TLR-2, TLR-3, TLR-4, TLR-7, and TLR-8, their expression of TLR-6 is elevated in comparison with PAMM1a and PAMM1b. TLR-9 expression is low to negative in HBCs, PAMM1a, and PAMM1b. Interestingly, TLR expression is poorly captured by scRNA-seq (Fig. S3 E), highlighting potential issues with overreliance on gene expression data alone.

**Figure S4.**
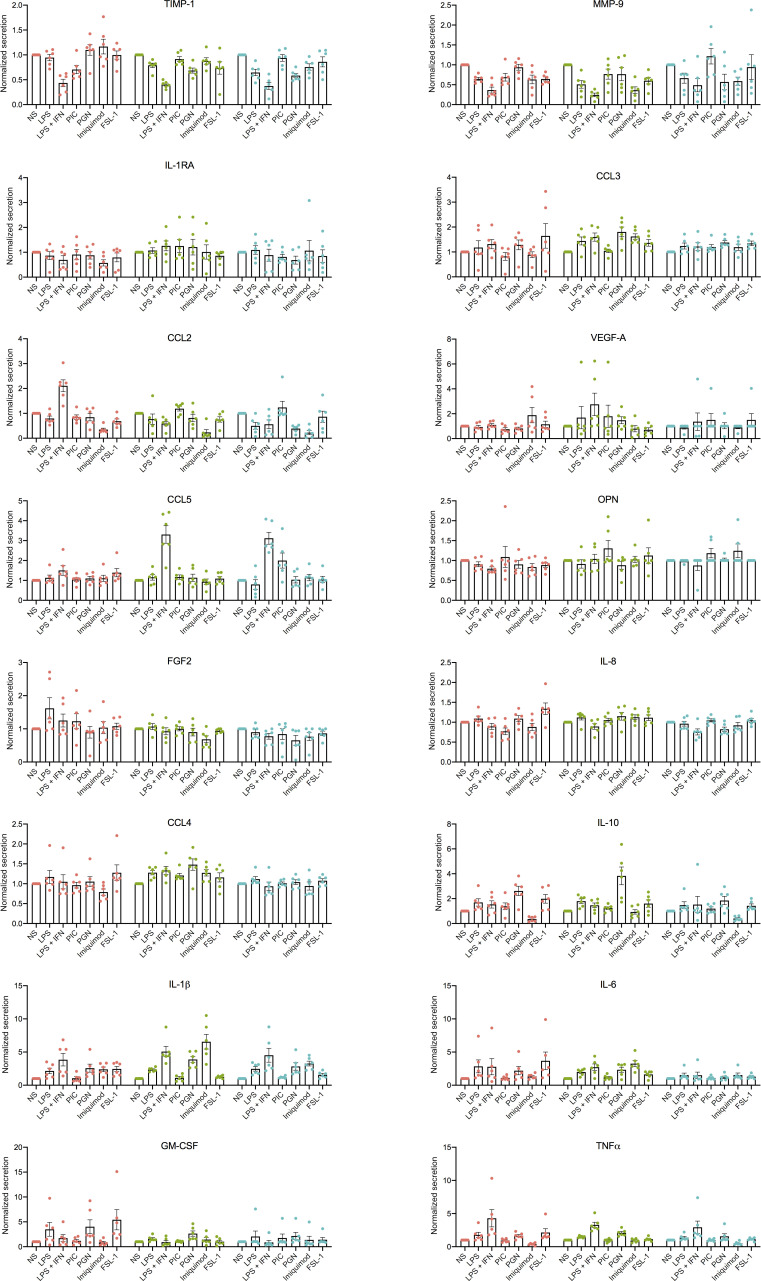
**HBC, PAMM1a, and PAMM1b secretome analysis in response to TLR stimulation.** Normalized cytokine, chemokine, and growth factor secretion of FACS-isolated HBCs, PAMM1a, and PAMM1b after 18 h in culture with TLR stimulation, relative to without stimulation (HBC, red; PAMM1a, green; PAMM1b, cyan). Profiled by Luminex (*n* = 6). Data are represented as mean ± SEM. NS, no stimulation control; PGN, peptidoglycan; P.I.C., polyinosinic-polycytidylic acid.

We next determined the response of HBCs, PAMM1a, and PAMM1b to TLR stimulation by analyzing their production of cytokines and growth factors after overnight stimulation with TLR agonists. Due to differences between subsets in their baseline expression of cytokines and growth factors, as demonstrated in Fig. 5 A, expression levels are normalized to unstimulated controls (Fig. 5 E and Fig. S4). The response of HBCs to TLR stimulation is specific to the agonist used, with LPS + IFNγ and FSL-1, a TLR-6 agonist, having the greatest impact. A combination of LPS and IFNγ impairs the ability of HBCs to produce factors important in tissue remodeling, including TIMP-1 and MMP-9, and increases the secretion of IL-1β and TNFα. FSL-1 increases HBC production of CCL-3, IL-8, IL-6, and GM-CSF. In contrast, PAMM1a and PAMM1b did not respond to FSL-1. These data show that primitive HBCs are capable of recognizing and responding to microbial stimulation and highlight the distinct responses of HBCs compared with maternal PAMM1a and PAMM1b.

### HBCs demonstrate microbicidal capacity

Given that breaks in SCT could provide a placental entry point for microbes and HBCs are responsive to TLR stimulation, we next sought to determine if HBCs have the mechanisms in place to kill microbes. Although mechanisms used by adult macrophages to kill microbes are well described in the literature, it remains unclear if primitive macrophages such as HBCs can use these.

**Figure S5.**
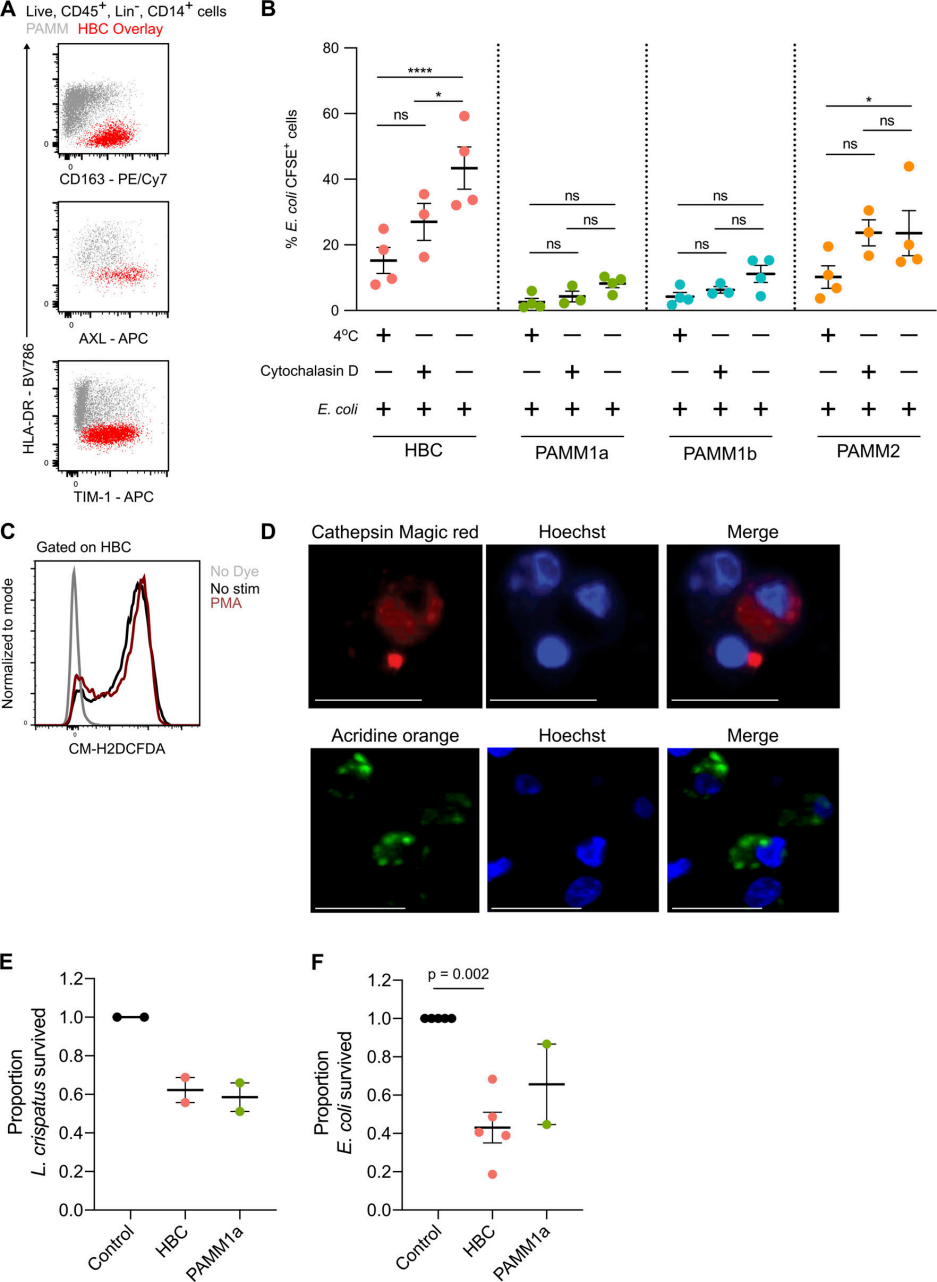
**Phagocytic and antibacterial capacity of HBCs and PAMM1a.** **(A)** Flow cytometric plots of scavenger receptor expression in HBCs (red) and PAMMs (gray). Representative plots of *n* = 3 experiments. **(B)** Phagocytosis of CFSE-labeled *E. coli* by HBC, PAMM1a, PAMM1b, and PAMM2 subsets measured by flow cytometry. P values were calculated by two-way ANOVA with Tukey’s multiple-comparisons test (*n* ≥ 3). **(C)** Representative flow cytometric plot of CM-H2DCFDA staining in FACS-isolated HBCs with no stimulation (black) and with PMA (red) relative to no stain (gray; representative plot from *n* = 3 experiments). **(D)** Cathepsin B activity, determined by cathepsin B Magic Red staining, and AO staining of lysosomes in FACS-isolated PAMM1a co-cultured with zymosan particles. Scale bars, 20 µm. Representative images of *n* = 3 experiments. **(E and F)** Rates of *L. crispatus* (E) *and E. coli* (F) killing by HBCs and PAMM1a after 1 h co-culture at an MOI of 10 relative to negative control, where no cells were added. P values were calculated by one sample *t* test (*n* ≥ 2). Data are represented as mean ± SEM. ns, not significant (P > 0.05); *, P ≤ 0.05; ****, P ≤ 0.0001.

**Figure 6.**
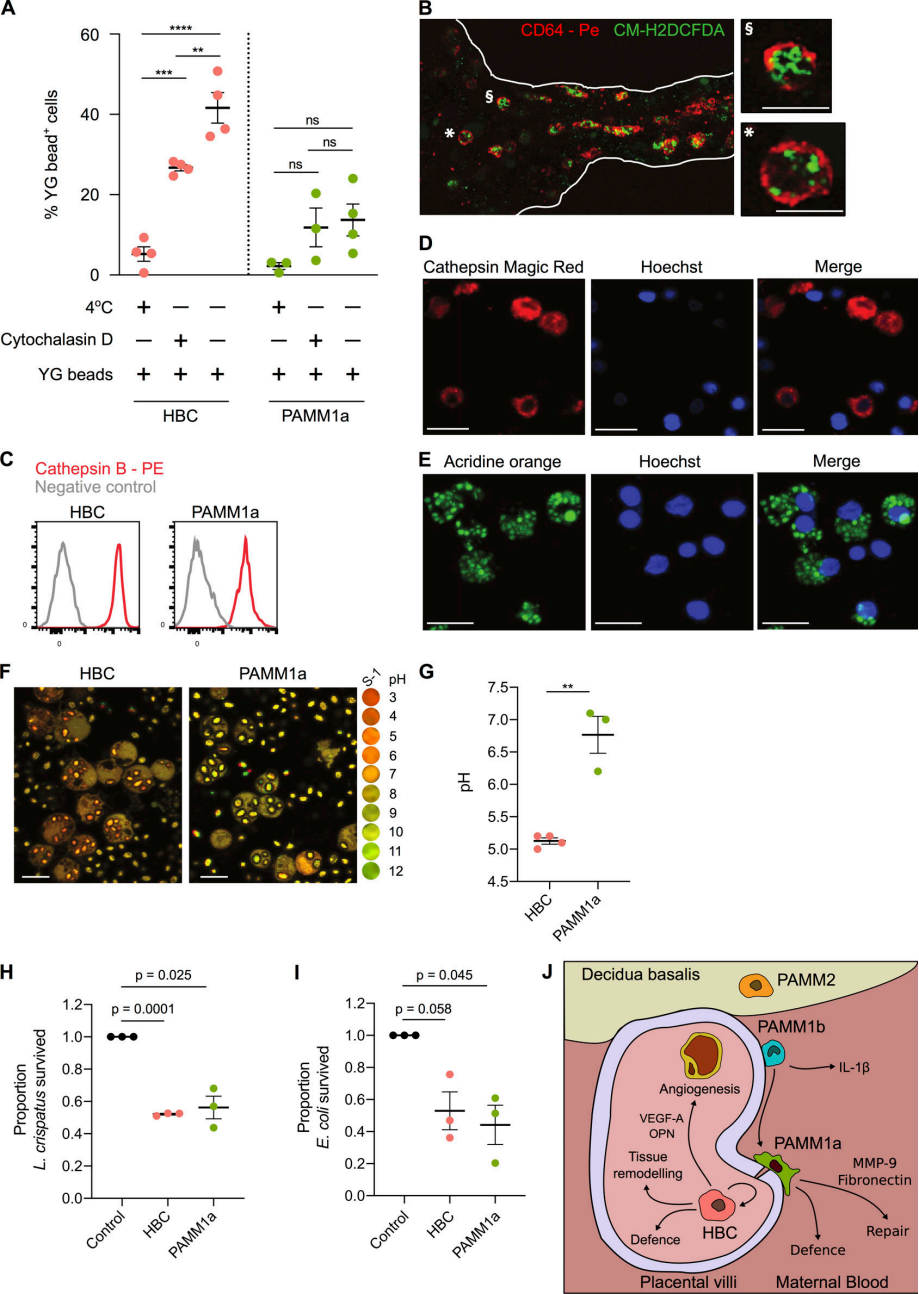
**HBCs are capable of mounting a microbicidal response.** **(A)** Phagocytosis of Fluoresbrite Yellow Green Microspheres (YG beads) by FACS-isolated HBC and PAMM1a measured by flow cytometry. P values were calculated by two-way ANOVA with Tukey’s multiple-comparisons test (*n* ≥ 3). **(B)** Whole-mount immunofluorescence microscopy of a placental villus showing CD64 expression (red) and ROS-dependent probe CM-H2DCFDA (green). Edges of villus are indicated by white lines. Right panels show magnification of individual cells, denoted by symbols. Scale bars, 20 µm. Representative image of *n* = 3 experiments. **(C)** Relative expression of cathepsin B in HBCs and PAMM1a to FMO control (gray), measured by flow cytometry (*n* = 3). **(D)** Cathepsin B activity in FACS-isolated HBCs, co-cultured with zymosan particles, determined by cathepsin B Magic Red staining. Scale bars, 20 µm. Representative images of *n* = 3. **(E)** AO staining of lysosomes in FACS-isolated HBCs co-cultured with zymosan particles. Scale bars, 20 µm. Representative images of *n* = 3 experiments. **(F and G)** Comparison of the phagosomal pH of HBCs and PAMM1a. **(F)** Representative images of phagosomal pH of FACS-isolated HBCs and PAMM1a after co-culture with Carboxy S-1–labeled zymosan particles for 20 min. The cytosol is labeled with 5- (and 6-) carboxy S-1 acetoxymethyl (S-1-AM) ester, a cell-permeant pH indicator. Right panel shows the pH scale. **(G)** Quantification of phagosomal pH; each data point represents an average of >100 measurements per separate donor (*n* ≥ 3). P value was calculated by unpaired *t* test. **(H and I)** Rates of *L. crispatus* (H) and *E. coli* (I) killing by HBCs and PAMM1a after 1 h co-culture at an MOI of 1, relative to negative control, where no macrophages were added. P values were calculated by a one-sample *t* test (*n* = 3). **(J)** Schematic depicting locations and subset-specific roles of placental macrophages. Data are represented as mean ± SEM. ns, not significant (P > 0.05); **, P ≤ 0.01; ***, P ≤ 0.001; ****, P ≤ 0.0001.

HBCs highly express receptors involved in phagocytosis, including CD64 (binds to IgG immune complexes), the mannose receptor CD206 (Fig. 1 G), and the scavenger receptors CD163, AXL, and TIM1 (recognizes phosphatidylserine and is critical for the uptake of apoptotic cells; Kobayashi et al., 2007; Fig. S5 A). In line with these findings, HBCs display increased phagocytic capacity of Fluoresbrite Yellow Green Microspheres (YG beads; Fig. 6 A) and CFSE-labeled *Escherichia coli* (Fig. S5 B) in comparison with PAMM1a. Cells cultured at 4°C and in the presence of cytochalasin D (an inhibitor of actin polymerization) were used as controls for beads bound to the cellular surface.

During phagocytosis, phagosomes fuse with lysosomes, resulting in production of reactive oxygen species (ROS) and protease activation. We tested if HBCs can produce ROS using the ROS indicator CM-H2DCFDA. Isolated HBCs make ROS even without PMA stimulation (Fig. S5 C), possibly due to the stress of the cell isolation protocol. Using ex vivo whole-mount immunofluorescence microscopy on placental explants, we find that the CD64^+^ HBCs in the villous stroma produce ROS, as indicated by CM-H2DCFCA staining (Fig. 6 B).

Both HBCs and PAMMs also express high levels of the protease cathepsin B (Fig. 6 C). Cathepsin B is active within HBCs and PAMM1a, as demonstrated using cathepsin Magic Red (Fig. 6 D and Fig. S5 D). HBCs and PAMM1a also contain lysosomal structures that were identified by the addition of acridine orange (AO; Fig. 6 E and Fig. S5 D). An acidic environment in phagosomes directly aids in bacterial killing and is also important for the activation of pH-sensitive antimicrobial enzymes (Sedlyarov et al., 2018; Flannagan et al., 2015). To determine if the HBC phagosome becomes acidic during maturation, we profiled the uptake of zymosan particles tagged with a pH-sensitive probe (Carboxy SNARF-1 [S-1]; Foote et al., 2017, 2019). After allowing phagocytosis for 20 min, we find that the HBC phagosome becomes rapidly acidic, reaching a pH of ∼4.5 (Fig. 6, F and G). In contrast, the phagosomes of PAMM1a are more alkaline, with a pH of ∼7.4 (Fig. 6, F and G). The pH of the PAMM1a phagosome is characteristic of antigen-presenting cells that are processing peptides for presentation (Savina et al., 2006).

Finally, to confirm that first-trimester HBCs have microbicidal capacity, we cultured HBCs with *Lactobacillus crispatus* (a microbe reported to be found in very low abundance within the second trimester fetal intestine; Rackaityte et al., 2020) and *E. coli* (not found in the fetus; Rackaityte et al., 2020). We find that HBC are as efficient as PAMM1a at killing both *L. crispatus* and *E. coli* when cultured at a multiplicity of infection (MOI) of 1 (Fig. 6, H and I) and 10 (Fig. S5, E and F).

Together, these data demonstrate that HBCs, a population of primitive macrophages, exhibit a range of microbicidal tools and have the capacity to kill bacteria in vitro.

## Discussion

Here, we describe methods for the identification, isolation, and characterization of first-trimester HBCs. We have summarized our key findings and conclusions in Fig. 6 J. Through the application of multiparameter flow cytometry, anti-HLA antibodies, and analysis of publicly available scRNA-seq datasets, we find that CD14^+^ cells obtained from first-trimester placental digests contain both fetal macrophages and maternal monocytes/macrophages. Our results indicate that all previous findings on HBCs from placental digests will include these maternal myeloid cells. PAMMs constitute ∼20–40% of isolated CD14^+^ cells and consist of three populations: PAMM1a, PAMM1b, and PAMM2. PAMM1a’s are maternal monocyte-derived macrophages that have adopted a phenotype specific to the placental niche. PAMM1b’s are very similar to maternal monocytes but display elevated expression of 150 genes when compared with matched maternal blood monocytes. This may reflect an adaptation to their location in the intervillous space, a unique microenvironment that can attract specific immune cells during gestation (Solders et al., 2019). However, these differences in gene expression could also reflect the isolation process for placental cells. Given their similar phenotype, PAMM2s are likely to be decidual macrophages, previously termed dMac2 (Vento-Tormo et al., 2018), that contaminate the cell isolates from uterine/placental tissues from early pregnancy. They are relatively rare cells, and their properties were not studied further.

Our data demonstrate that HBCs are a homogenous population of cells that are transcriptionally similar to primitive YS macrophages, further emphasizing their origin through primitive hematopoiesis. While fate-mapping studies using murine models have determined the origins of many tissue macrophage populations (Ginhoux and Guilliams, 2016), the origin of HBCs has yet to be elucidated. The placenta is a known site of primitive hematopoiesis (Van Handel et al., 2010), but whether this occurs independently of the YS or YS macrophages migrate to the placenta, giving rise to HBCs, is unknown. HBCs, YS macrophages, and YS-derived macrophages from a CS10 embryo do not express HLA-DR. This suggests that the lack of HLA-DR is an intrinsic property of primitive macrophages and could be used to distinguish macrophages derived from primitive and definitive hematopoiesis in other fetal tissues.

Previous studies have yielded variable findings concerning the phenotype, cytokine secretion, and functions of HBCs (Johnson and Chakraborty, 2012; Young et al., 2015; Pavlov et al., 2020; Loegl et al., 2016; Schliefsteiner et al., 2017; Swieboda et al., 2020), probably due to the failure to account for PAMM contamination, which we show is present in placental digests. Here, using our gating strategy for the isolation of placental myeloid cell populations, we find that steady-state HBCs secrete a range of factors that play a role in vascularization and the remodeling of blood vessels, such as VEGF-A, OPN, MMP-9, and TIMP-1 (Johnson et al., 2003; Dai et al., 2009; Poggio et al., 2011; Luizon et al., 2014; Plaks et al., 2013). HBCs also secrete factors that are typically associated with inflammation, including IL-8, CCL-2, CCL-3, and CCL-4. IL-8 is a potent neutrophil chemoattractant (Hammond et al., 1995), but neutrophils are absent from the healthy placenta. In the context of the placenta, it is therefore likely that these factors are proangiogenic. For example, in vitro assays (Shi and Wei, 2016) using a physiological range of IL-8 (0.2–1 ng/ml; we found that HBCs produce ∼128 ng/ml/10^4^ cells of IL-8), have shown that IL-8 promotes the migration and canalization of HUVECs and their production of VEGF-A (Shi and Wei, 2016).

The SCT layer that covers the placental surface always contains sites of damage during healthy pregnancy (Costa et al., 2017; Burton and Watson, 1997), particularly at bridges between two villi (Burton and Watson, 1997). Fibrin deposits are typically present at the sites of breaks in the syncytium (Burton and Watson, 1997). We suggest that PAMM1a’s are the macrophages that have been identified at sites of damage at the syncytium (Burton and Watson, 1997) and are mediators of the repair process, as they adopt a tissue-repair phenotype and are transcriptionally similar to SAMac in human liver fibrosis. PAMM1a’s are also laden with lipid droplets and are deficient in phagocytosis. This finding is resonant with data showing microglia laden with lipid droplets also display impaired phagocytosis (Marschallinger et al., 2020), although the responsible mechanisms are still unknown. PAMM1a may have reached a point of “saturation” through the uptake of cellular debris at the placental surface, reducing further phagocytosis. In future studies, it will be interesting to determine the role of PAMM1a in pregnancy disorders, including preeclampsia and transplacental infection.

Our work shows that while HBCs are primitive macrophages in terms of origin, they are not primitive in function, as demonstrated by their response to TLR stimulation and their microbicidal capacity. The distinct response of HBCs to TLR stimulation in comparison with PAMM1a reflects their TLR expression profile. For example, HBCs highly express TLR-6 (which binds to bacterial lipoproteins) and strongly respond to TLR-6 stimulation in comparison with PAMM1a. The elevated expression of TLR-6 by HBCs is surprising given its expression is restricted to a select number of human tissues, such as the spleen (Fagerberg et al., 2014). While HBCs demonstrate increased phagocytic capacity and adopt a more acidic phagosome in comparison with PAMM1a, PAMM1a’s are as efficient as HBCs at killing both *E. coli* and *L. crispatus*. This equivalent microbicidal capacity can be explained by active cathepsin B activity and other antimicrobial mechanisms that PAMM1a may have. Given the microbicidal capacity of HBCs, it is of interest to study their interaction with microbes that do cross the placental barrier and cause an adverse pregnancy outcome, such as *Listeria monocytogenes* and Zika virus. However, these areas of research were beyond the scope of this study.

The work presented in this study is limited to first-trimester samples. Previous studies have investigated the properties of HBCs across gestation (Goldstein et al., 1988; Ingman et al., 2010; Swieboda et al., 2020; Pavlov et al., 2020). However, studies that used placental digests have not considered contamination with PAMM populations, and so interpretation of some of their findings is difficult. Using the methods described here for the isolation and study of HBCs, an area of interesting future research will be to investigate how HBC phenotype and functions change throughout pregnancy.

In summary, we have provided a gating strategy that allows the study of human HBCs. We have inferred the roles of these cells at the steady-state and demonstrated the microbicidal capacity of human primitive macrophages. This study adds to our understanding of human developmental immunology and provides an important framework for the field of placental biology. Future studies will now aim to determine the roles of primitive HBCs in health and disease.

## Materials and methods

### Patient samples

All tissue samples used were obtained with written consent from participants. Decidual and placental tissues were obtained from healthy women with apparently normal pregnancies undergoing elective first-trimester terminations (6–12 wk EGA; *n* = 20). Peripheral blood was taken from women undergoing elective first-trimester terminations (6–12 EGA). The EGA of the samples was determined from the last menstrual period. All samples were obtained with written informed consent from participants under ethical approval, which was obtained from the Cambridge Research Ethics Committee (study 04/Q0108/23).

### Tissue processing

Placental samples were processed immediately upon receipt. Samples were washed in PBS for 10 min with a stirrer before processing. The placental villi were scraped from the chorionic membrane with a scalpel and digested with 0.2% Trypsin (Pan-Biotech)/0.02% EDTA (Source BioScience) at 37°C with stirring, for 7 min. The digested cell suspension was passed through a sterile muslin gauze, and FBS (Sigma-Aldrich) was added to halt the digestion process. The undigested tissue left on the gauze was scraped off with a scalpel and digested in 1 mg/ml collagenase V (Sigma-Aldrich), supplemented with 10 mg/ml DNase I (Roche) for 20 min at 37°C with agitation. The digested cell suspension was passed through a sterile muslin gauze and washed through with PBS. Cell suspensions from both the trypsin and collagenase digests were pelleted, resuspended in PBS and combined. Cells were layered onto a Pancoll gradient (PAN-biotech) and spun for 20 min without brake at 3,000 rotations per minute. The leukocyte layer was collected and washed in PBS. Decidual samples and blood were processed as described previously (Huhn et al., 2020).

### Flow cytometry

Cell suspensions were stained for viability with either 1:3,000 DAPI (Sigma-Aldrich) or 1:1,000 Zombie Aqua (BioLegend) for 20 min at 4°C and washed twice in PBS. Cells were blocked in human blocking buffer (5% human serum [Sigma-Aldrich], 1% rat serum [Sigma-Aldrich], 1% mouse serum [Sigma-Aldrich], 5% FBS, and 2 mM EDTA) for 15 min at 4°C and incubated with antibody cocktails for 30 min at 4°C. Antibodies used are listed in Table S1. Cells were washed and resuspended in FACS buffer (PBS containing 2% FBS and 2 mM EDTA). For intracellular staining, cells were fixed and permeabilized with BD PharMingen Transcription Factor Buffer (BD Biosciences) according to manufacturer’s instructions. The lineage channel in flow cytometry analyses included the markers CD3, CD19, CD20, CD66b, and CD335 for the removal of contaminating maternal T cells, B cells, natural killer cells, and granulocytes. Flow cytometry was performed using a Cytek Aurora (Cytek), or cells were purified by cell sorting using a BD FACS Aria III (BD Biosciences). All flow cytometry data were analyzed using FlowJo v10.6.1 (Tree Star).

### Whole-mount immunofluorescence microscopy

Biopsies of placental tissue (2 cm^3^) were prepared as described previously (Wang et al., 2014). Placental villi were blocked with microscopy blocking solution (1% BSA [Sigma-Aldrich] and 0.25% Triton X-100 [Sigma-Aldrich] in PBS) for 15 min and stained with antibodies (Table S1) suspended in microscopy blocking solution in 1.5-ml Eppendorf tubes for 1 h at room temperature or overnight at 4°C. The nuclear dye Hoechst 33342 (Abcam; diluted 1:2,000 in PBS) was added for 30 min before imaging. Whole mounts were mounted in a chamber system (Pecon; POC-R2 cell cultivation system). Imaging was performed using a Zeiss SP8 confocal LSM 700.

### Electron microscopy

Correlative scanning and transmission electron microscopy images of PAMMs on the surface of first-trimester placental villi were generated as previously described (Burton, 1986).

### Immunofluorescence of placental tissue sections

First-trimester placenta villous tissue and decidual sections were prepared as described previously. Slides were placed in blocking buffer for 20 min at room temperature, washed in PBS, and incubated overnight at 4°C with antibodies (Table S1). The slides were washed twice for 5 min in PBS and, when necessary, incubated with secondary antibodies for 1 h at room temperature and washed twice for 5 min in PBS. The slides were then air-dried and mounted using VECTASHIELD Antifade Mounting Medium with DAPI (Vector Laboratories). Slides were imaged using a Zeiss SP8 confocal LSM 700 (Zeiss).

### Immunohistochemistry

Slides were prepared as described previously (Sharkey et al., 1999). Antibodies used are indicated in Table S1. Slides were imaged on an EVOS M5000 microscope (Thermo Fisher Scientific).

### BODIPY staining of placental cells

Placental cells were stained for flow cytometry as described above. Cells were incubated in 2 ng/ml BODIPY 493/503 (Thermo Fisher Scientific) in PBS for 20 min at 4°C. Cells were washed in FACS buffer and acquired on a Cytek Aurora (Cytek).

FACS-isolated PAMM1a and PAMM1b were incubated in 250 ng/ml BODIPY 493/503 (Thermo Fisher Scientific) in PBS for 1 h at 37°C, fixed in 4% paraformaldehyde solution (Sigma-Aldrich), and washed twice in PBS. Cytospins were prepared and mounted using VECTASHIELD Antifade Mounting Medium with DAPI (Vector Laboratories) and imaged using a Zeiss SP8 confocal LSM 700.

### EdU incorporation assay

FACS-purified HBCs were plated at a density of 10,000 cells in 100 μl DMEM (Thermo Fisher Scientific) supplemented with 10% FBS, 2.5% penicillin streptomycin (Sigma-Aldrich), and 20 µM L-glutamine (Sigma-Aldrich). EdU incorporation was determined using the Click-IT Plus EdU Alexa Fluor 647 Flow Cytometry Assay Kit (Thermo Fisher Scientific), according to manufacturer’s instructions. Cells were incubated for 18 h before harvesting and acquisition by flow cytometry.

### Cytokine production and TLR stimulations

FACS-purified HBCs, PAMM1a, and PAMM1b were plated into V-bottom 96-well plates at a density of 10,000 cells in 50 µl DMEM supplemented with 10% FBS, 2.5% penicillin streptomycin, 20 µM L-glutamine, and 100 µM β2-mercaptoethanol (Sigma-Aldrich). Cells were incubated for 18 h without stimulus or with the following stimuli: 100 ng/ml LPS (Invivogen), 1,000 U/ml IFNγ (Novaprotein), 25 µg/ml polyinosinic:polycytidylic acid (InvivoGen), 20 µg/ml Imiquimod (Insight Technology), 10 µg/ml peptidoglycan (InvivoGen), and 200 ng/ml Pam2CGDPKHPKSF (FSL-1; InvivoGen). After incubation, plates were spun to remove cellular debris, and supernatants were collected and stored at −80°C.

Cell culture supernatants were tested for the presence of 16 analytes using a custom 10-plex Luminex ProcartaPlex assay (Thermo Fisher Scientific) and a custom 6-plex Luminex ProcartaPlex assay (Thermo Fisher Scientific) designed to profile the expression of CCL-2, CCL-3, CCL-4, CCL-5, FGF-2, GM-CSF, IL-1β, IL-1RA, IL-6, IL-8, IL-10, MMP-9, OPN, TIMP-1, TNFα, and VEGF-A. Samples were diluted in cell culture medium at a ratio of 1:1 for the 10-plex Luminex ProcartaPlex assay and 1:40 for the 6-plex Luminex ProcartaPlex assay. The Luminex assays were performed according to the manufacturer’s instructions, and beads were run on a Luminex LX-200 (Luminex) using xPONENT software (Luminex). Results were visualized using Prism 8 (GraphPad) and R version 3.5.1 (R Foundation).

### Phagocytosis assays

For the microsphere phagocytosis assay, macrophages were placed in 1.5-ml Eppendorfs at 10,000 cells in 100 µl PBS, and human serum opsonized Fluoresbrite Yellow Green Microspheres (1 µm; Polysciences) were added at a concentration of 10:1 for 1 h. Controls included cells cultured at 4°C and 37°C in the presence of 10 µM cytochalasin D (Sigma-Aldrich).

For the *E. coli* phagocytosis assay, *E. coli* were grown until log-phase growth and an optical density (595 nm) of 0.3. Bacteria were opsonized in heat-inactivated human serum for 30 min at 37°C and labeled via incubation with 10 µM CFSE (BioLegend) for 30 min at 37°C. Labeled bacteria were washed three times in PBS before use. First-trimester placental cell suspensions were plated at a density of 10^6^/ml per well in PBS. Labeled *E. coli* were added at an MOI of 10 and cultured for 1 h at 37°C. Control wells were incubated at 4°C and at 37°C in presence of 10 µM cytochalasin D. Plates were centrifuged at 200*g* for 5 min to promote cell–bacteria interactions. Cells were washed three times in 4°C PBS and stained for flow cytometry as described above.

### ROS production assays

FACS-isolated HBCs were plated at a density of 50,000 cells in 50 µl DMEM (Gibco) supplemented with 10% FBS (Sigma-Aldrich), 2.5% penicillin streptomycin, and 20 µM l-Glutamine. Cells were stained with 1 µM CM-H2DCFDA (Thermo Fisher Scientific) and were either treated with 1× cell activation cocktail (PMA-ionomycin; BioLegend) for 30 min or incubated without stimulation. Cells were washed in PBS and data acquired as described above.

Ex vivo imaging of HBC ROS production was performed by incubating placental villi with anti-CD64 antibody conjugated to PE and 1 µM CM-H2DCFDA for 15 min. The villi were placed in an Ibidi µ-Dish (35 mm) and imaged using a Zeiss SP8 confocal LSM 700.

### Cathepsin B activity and AO assays

Macrophages were seeded at 10,000 cells/well in 10 µl PBS on poly-L-lysine (Sigma-Aldrich)–coated Ibidi 4-well µ-Dish plates. Zymosan bioparticles (Thermo Fisher Scientific) were added at a concentration of 10 particles per cell. Cathepsin B activity was determined using Magic Red (a cell-permeable and noncytotoxic reagent that contains a cathepsin B target sequence peptide [RR]2 linked to a red [Cresyl Violet] fluorescent probe) and lysosomes were detected with AO (Bio-Rad), according to the manufacturer’s instructions.

### Phagosomal pH assay

Placental macrophage phagosomal pH measurements were performed by adapting a method using the fluorescent-sensitive pH dye S-1 with a dual-emission spectrum (Foote et al., 2017). Carboxy S-1-succinimidyl ester (Thermo Fisher Scientific) was coupled to zymosan coated beads (Thermo Fisher Scientific) and opsonized with human serum. Macrophages were cultured at 10,000 cells/well in 10 µl PBS, in poly-lysine (Sigma) coated Ibidi 4-well μ-Dish plates. 5 × 10^5^ S-1–labeled beads were added per well to macrophages. 50 µg of carboxy S-1-acetoxymethyl (S-1-AM) ester (Thermo Fisher Scientific) was added as a cytosolic dye (0.5 µg/ml working solution). Subsequently, cells were examined under a 63× oil-immersion objective on a Zeiss SP8 confocal LSM 700, where cells were excited at 555 nm and emission was measured at 560–600 nm and 600–610 nm. Over 100 measurements were made per condition. The pH scale was generated as described previously (Foote et al., 2017).

### *E. coli* and *L. crispatus* killing assays

*E. coli* and *L. crispatus* were grown overnight in Luria-Bertani broth and Man, Rogosa, and Sharpe broth, respectively. Bacteria were subcultured the following day until they had reached log-phase growth and an optical density (595 nm) of 0.3. Bacteria were opsonized in heat-inactivated human serum for 30 min at 37°C. FACS-purified placental cells were plated into 96-well plates at a density of 10,000 cells in 50 µl DMEM (Gibco) supplemented with 10% FBS (Sigma-Aldrich), 20 µM L-glutamine, and 25 mM Hepes (Gibco). Bacteria were added at an MOI of 1 or 10, and plates were centrifuged at 200*g* for 5 min to promote cell–bacteria interactions. After incubation for 1 h, cells were lysed in deionized water, and serial dilutions were plated onto Luria-Bertani agar or Man, Rogosa, and Sharpe agar plates for *E. coli* and *L. crispatus* experiments, respectively. CFUs were counted after 24 h for *E. coli* and after 48 h for *L. crispatus*.

### Analysis of publicly available scRNA-seq data

scRNA-seq data of first-trimester placenta (Vento-Tormo et al., 2018) were obtained at from EMBL-EBI ArrayExpress (https://www.ebi.ac.uk/arrayexpress) under the experiment code E-MTAB-6701. Sequencing data from placental samples were aligned using the Cell Ranger Single-Cell Software Suite (v3.0; 10x Genomics) against the GRCh38.93 human reference genome. Downstream analysis of each sample was performed using Seurat (v3.0; Butler et al., 2018). Cells with <500 detected genes and >20% mitochondrial gene expression were removed. Samples were log-normalized and integrated following the Seurat v3 Integration workflow. Clusters were identified using the FindNeighbors and FindClusters functions in Seurat. Clusters were annotated on the basis of expression of known marker genes. UMAP dimensionality reduction was performed using the RunUMAP function in Seurat, with default parameters. Significantly DEGs were identified using the FindMarkers function, using the Wilcoxon rank sum test, corrected for multiple comparisons.

scRNA-seq data from early human fetal immune cells were obtained from the Gene Expression Omnibus (GEO) database under the accession code GSE133345. The dataset was analyzed using Seurat and subset to include only myeloid cells. These cells were integrated with HBC and PAMM clusters from the placenta scRNA-seq dataset using the reference-based integration workflow in Seurat, using the early human fetal myeloid cell object as a reference. Pearson’s correlations between annotated clusters were calculated using the average expression per cluster of the 2,000 variable genes used for integration. Cell cycle scoring of HBCs was performed using the CellCycleScoring function in Seurat, and predicted cell cycle states were overlaid onto UMAP embeddings.

Cell Ranger output files for each sample were analyzed using Velocyto (La Manno et al., 2018; python version 0.17.17). Output loom files were merged with Seurat objects in R, and RNA velocity vectors were calculated using the RunVelocity function from the SeuratWrappers package and projected onto UMAP embeddings using the show.velocity.on.embedding.cor function from the VelocytoR package.

Comparisons of cell-type similarity between datasets was performed using a random forest model in the ranger R package, as previously described (Stewart et al., 2019). Peripheral blood mononuclear cell (PBMC) scRNA-seq data for comparison was downloaded from 10x Genomics (https://support.10xgenomics.com/single-cell-gene-expression/datasets). Expression matrices from both datasets were subset using the union of the highly variable features detected in each dataset. The random forest model was built using the ranger function on the PBMC dataset, and single-cell prediction scores were generated for placental cells using the predict function.

Single-cell gene signature enrichment scores were calculated using the AddModuleScore function in Seurat. Gene signatures from the cirrhotic liver SAMac and Kupffer cells (KCs; Ramachandran et al., 2019) were generated from the analysis of scRNA-seq data obtained from GEO under the accession code GSE136103. In brief, the datasets were aligned and preprocessed as described above and subset to include only the myeloid compartment. DEGs in SAMac and KC clusters were identified, and genes with log fold change >0.5 and adjusted P value <0.05 were used as gene signatures. DEGs were identified between YS macrophages and embryonic monocytes (Bian et al., 2020), and the top 50 genes with log fold change >0.5 and adjusted P value <0.05 were used as the gene signatures for each population. The gene signature from CS10 macrophages (Zeng et al., 2019) was generated from the analysis of scRNA-seq data obtained from GEO under the accession code GSE135202. The data were processed as described above and subset to include endothelial and hematopoietic populations. DEGs in macrophages were identified, and the top 50 genes with log fold change >0.5 and adjusted P value <0.05 were used as the gene signature.

Pseudotime trajectory analysis of PAMMs was performed with the Slingshot R package (Street et al., 2018), and the calculated trajectory was overlain onto the UMAP embeddings. Selected genes that varied across the Slingshot trajectory were plotted as heatmaps of smoothed scaled gene expression. Smoothing was performed using the rollmean function in the zoo R package.

### Cell–cell interaction analyses

Predicted cell–cell ligand–receptor interactions were inferred from scRNA-seq data using CellphoneDB (Efremova et al., 2020) using the online tool (https://www.cellphonedb.org). The minimum proportion of cells in a cluster expressing a gene was set to 10%, and the number of iterations was set to 1,000. Ligand–receptor pairs were subset to include only the 16 ligands profiled in Luminex experiments. An estimate for interaction potential between cells was obtained by multiplying the log-normalized cytokine secretion of each ligand from sort-purified cell populations by the average log-normalized gene expression of each receptor in each cluster.

### Online supplemental material

Fig. S1 includes a schematic illustrating the protocol used to obtain single-cell suspensions from placental tissue and HLA allotype staining for matched maternal blood and decidua. Fig. S2 shows the identification and characterization of PAMM populations. Fig. S3 shows the data for cytokine, chemokine, and growth factor secretion. Fig. S4 shows the normalized cytokine, chemokine, and growth factor secretion. Fig. S5 shows the phagocytic and microbial capacity of HBCs and PAMM1a. Data S1 presents the gene signatures used for scRNA-seq enrichment analyses (related to Fig. 2, A and D; and Fig. 4 J). Table S1 provides a list of reagents, antibodies, and software used in this study.

## Supplementary Material

Table S1 shows the antibodies, reagents, and software used in this study.

Data S1 presents the gene signatures used for scRNA-seq enrichment analyses.
