# Supplementary material for: Phenotypic and functional characterization of first-trimester human placental macrophages, Hofbauer cells
Source: J Exp Med. 2020 Oct 19;218(1):e20200891. doi: 10.1084/jem.20200891 (PMC7579740; doi:10.1084/jem.20200891)
Supplement: Table S1 — shows the antibodies, reagents, and software used in this study. [file JEM_20200891_TableS1.docx]

Table S1. Antibodies, reagents, and software used

| Reagent or resource | Source | Identifier |
| --- | --- | --- |
| **Antibodies** | | |
| Anti-human arginase 2 (clone poly) | Abcam | Catalog no. ab81505, RRID: AB_1861680 |
| Anti-human AXL (APC; clone 108724) | R&D Systems | Catalog no. FAB154A, no RRID |
| Anti-human cathepsin D (unconjugated; clone poly) | Thermo Fischer Scientific | Catalog no. PA5-17353, RRID: AB_10981418 |
| Anti-human CCR2 (BV421; clone K036C2) | BioLegend | Catalog no. 357209, RRID: AB_2562293 |
| Anti-human CCR2 (PE; clone K036C2) | BioLegend | Catalog no. 357205, RRID: AB_2562058 |
| Anti-human CD14 (PE/Dazzle; clone HCD14) | BioLegend | Catalog no. 325634, RRID: AB_2563625 |
| Anti-human CD163 (PE/Cy7; clone GHI/61) | BioLegend | Catalog no. 333613, RRID: AB_2562640 |
| Anti-human CD19 (FITC; clone SJ25C1) | BioLegend | Catalog no. 363008, RRID: AB_2564171 |
| Anti-human CD20 (FITC; clone 2H7) | BioLegend | Catalog no. 302304, RRID: AB_314252 |
| Anti-human CD206 (PE; clone 15-2) | BioLegend | Catalog no. 321105, RRID: AB_571910 |
| Anti-human CD206 (PerCP/Cy5.5; clone 15-2) | BioLegend | Catalog no. 321121, RRID: AB_10900990 |
| Anti-human CD282 (TLR2; PE; clone TL2.1) | BioLegend | Catalog no. 309707, RRID: AB_314777 |
| Anti-human CD283 (TLR3; PE; clone TLR-104) | BioLegend | Catalog no. 315005, RRID: AB_2303469 |
| Anti-human CD284 (TLR4; PE; clone HTA125) | BioLegend | Catalog no. 312805, RRID: AB_314954 |
| Anti-human CD286 (TLR6; PE; clone TLR 6.127) | BioLegend | Catalog no. 334707, RRID: AB_2205398 |
| Anti-human CD289 (TLR9; PE; clone S16013D) | BioLegend | Catalog no. 394803,  RRID: AB_280103 |
| Anti-human CD3 (FITC; clone UCHT1) | BioLegend | Catalog no. 300406, RRID: AB_314060 |
| Anti-human CD31 (AF488; clone WM59) | BioLegend | Catalog no. 303109, RRID: AB_493075 |
| Anti-human CD335 (FITC; clone 9E2) | BioLegend | Catalog no. 331921, RRID: AB_2561964 |
| Anti-human CD36 (FITC; clone 5-271) | BioLegend | Catalog no. 336203, RRID: AB_1575029 |
| Anti-human CD365 (TIM-1; APC; clone 1D12) | BioLegend | Catalog no. 353905, RRID: AB_2564324 |
| Anti-human CD45 (BUV395; clone HI30) | BioLegend | Catalog no. 563792, RRID: AB_2744400 |
| Anti-human CD45 (BV605; clone HI30) | BioLegend | Catalog no. 304042, RRID: AB_2562106 |
| Anti-human CD45 (PerCP/Cy5.5; clone 2D1) | BioLegend | Catalog no. 368503, RRID: AB_2566351 |
| Anti-human CD56 (FITC; clone HCD56) | BioLegend | Catalog no. 318304, RRID: AB_604100 |
| Anti-human CD63 (AF647; clone H5C6) | BioLegend | Catalog no. 353015, RRID: AB_2561662 |
| Anti-human CD64 (BV605; clone 10.1) | BioLegend | Catalog no. 305033, RRID: AB_2566236 |
| Anti-human CD64 (PE; clone 10.1) | BioLegend | Catalog no. 305007, RRID: AB_314491 |
| Anti-human CD66b (AF700; clone G10F5) | BioLegend | Catalog no. 305113, RRID: AB_2566037 |
| Anti-human CD66b (FITC; clone G10F5) | BioLegend | Catalog no. 305103, RRID: AB_314495 |
| Anti-human CD68 (PE; clone Y1/82A) | BioLegend | Catalog no. 333807, RRID: AB_1089057 |
| Anti-human CD9 (FITC; clone HI9a) | BioLegend | Catalog no. 312103, RRID: AB_314908 |
| Anti-human CD9 (PE/cy7; clone HI9a) | BioLegend | Catalog no. 312115, RRID: AB_2728255 |
| Anti-human FOLR2 (APC; clone 94b/FOLR2) | BioLegend | Catalog no. 391705, RRID: AB_2721302 |
| Anti-human FOLR2 (PE; clone 94b/FOLR2) | BioLegend | Catalog no. 391703, RRID: AB_2721335 |
| Anti-human HLA-A2 (APC/Cy7; clone BB7.2) | BioLegend | Catalog no. 343310, RRID: AB_2561568 |
| Anti-human HLA-A3 (APC; clone GAP.A3) | Thermo Fischer Scientific | Catalog no. 17-5754-42, RRID: AB_2573220 |
| Anti-human HLA-A3 (BV650; clone GAP.A3) | BD Biosciences | Catalog no. 747774, RRID: AB_2739760 |
| Anti-human HLA-B7 (Biotin; clone REA176) | Miltenyi Biotec | Catalog no. 130-106-046, RRID: AB_2652117 |
| Anti-human HLA-B7 (PE; clone BB7.1) | BioLegend | Catalog no. 372403, RRID: AB_2650773 |
| Anti-human HLA-DR (APC; clone L243) | BioLegend | Catalog no. 307605, RRID: AB_314683 |
| Anti-human HLA-DR (BV711; clone L243) | BioLegend | Catalog no. 307643, RRID: AB_11218794 |
| Anti-human HLA-DR (BV786; clone G46 - 6) | BD Biosciences | Catalog no. 564041, RRID: AB_2738559 |
| Anti-human HLA-DR (FITC; clone G46 - 6) | BioLegend | Catalog no. 327006, RRID: AB_893569 |
| Anti-human HLA-DR (PE; clone L243) | BioLegend | Catalog no. 307609, RRID: AB_314687 |
| Anti-human Ki67 (PE; clone SolA15) | Thermo Fischer Scientific | Catalog no. 12569882, RRID: AB_11150954 |
| Anti-human Ki67 (unconjugated; clone Ki-67) | BioLegend | Catalog no. 350501, RRID: AB_10662749 |
| Anti-human LOX1 (BV421; clone 15C4) | BioLegend | Catalog no. 358609, RRID: AB_2728342 |
| Anti-human lysozyme (FITC; clone LZ-2) | Thermo Fischer Scientific | Catalog no. GIC207, RRID: AB_2536533 |
| Anti-human TLR7 (PE; clone 4G6) | Thermo Fischer Scientific | Catalog no. MA5-16249, RRID: AB_2537767 |
| Anti-human TLR8 (PE; clone 44C143) | Abcam | Catalog no. ab45097, RRID: AB_778508 |
| **Bacterial and viral strains** | | |
| *L. crispatus* | ATCC | ATCC-33820 |
| **Chemicals, peptides, and recombinant proteins** | | |
| DAB | Sigma-Aldrich | D4168-50SET |
| DAPI | Sigma-Aldrich | D9542 |
| Acetone | Sigma-Aldrich | 179124-1L |
| Aqua Zombie Fixable Viability Kit | BioLegend | 423101 |
| BD Difco dehydrated culture media: Lactobacilli MRS agar | Thermo Fischer Scientific | DF0882-17-0 |
| BD Difco Lactobacilli MRS Broth | Thermo Fischer Scientific | 11713553 |
| BD Pharmingen Transcription Factor Buffer | BD Biosciences | 562574 |
| β2-Mercaptoethanol | Sigma-Aldrich | 444203 |
| BODIPY 493/503 | Thermo Fisher Scientific | D3922 |
| BSA | Sigma-Aldrich | A9418 |
| Carazzi’s hematoxylin | Clin-Tech Ltd | 642305 |
| 5-(and 6-)-Carboxy S-1, acetoxymethyl ester, acetate | Thermo Fischer Scientific | C1272 |
| S-1 carboxylic acid, acetate, succinimidyl ester | Thermo Fischer Scientific | S22801 |
| CFSE | BioLegend | 423801 |
| Cell activation cocktail | BioLegend | 423301 |
| CM-H2DCFDA | Thermo Fischer Scientific | C6827 |
| Collagenase V | Sigma-Aldrich | C9263 |
| Cytochalasin D | Sigma-Aldrich | C8273 |
| DNase I | Roche | 10104159001 |
| Advanced DMEM/F-12 | Thermo Fisher Scientific | 12634028 |
| EDTA | Sigma-Aldrich | 324506 |
| FBS | Sigma-Aldrich | f9665-500ML |
| Fluoresbrite Yellow Green Microspheres, 1 μm | Polysciences | 17154 |
| Gibco Hepes (1 M) | Thermo Fischer Scientific | 11560496 |
| Giemsa stain | Sigma-Aldrich | 48900-500ML-F |
| Glycerol gelatin | Sigma-Aldrich | GG1-15ML |
| Hoechst 33342 dye | Abcam | ab228551 |
| Human AB serum | Sigma-Aldrich | H4522 |
| Ibidi 4-well m-Dish plates | Ibidi | 80406 |
| IFNγ | Thermo Fisher Scientific | PHC4031 |
| Imiquimod | InvivoGen | Tlrl-imqs |
| Invitrogen Zyomosan A Bioparticles | Thermo Fisher Scientific | Z2849 |
| L-Glutamine | Sigma-Aldrich | G7513 |
| *L. crispatus* | ATCC | ATCC-33820 |
| LPS | Invivogen | tlrl-b5lps |
| Methanol | Thermo Fischer Scientific | 10675112 |
| Mouse serum | Sigma-Aldrich | M5905 |
| Optimal cutting temperature embedding medium | Thermo Fisher Scientific | 12678646 |
| Pam2CGDPKHPKSF (FSL-1) | Invivogen | tlrl-fsl |
| Pancoll | Pan-Biotech | P04-60500 |
| Paraformaldehyde | Thermo Fisher Scientific | 43368 |
| Penicillin streptomycin | Sigma-Aldrich | P4333 |
| Peptidoglycan | Invivogen | tlrl-pgns2 |
| Poly-L-lysine | Sigma-Aldrich | P4707 |
| Poly(I:C) | Invivogen | tlrl-pau |
| Rat serum | Sigma-Aldrich | R9759-5ML |
| Triton X-100 | Sigma-Aldrich | X100-500ML |
| Trypsin | Pan-Biotech | P10-025100P |
| VECTASHIELD Antifade Mounting Medium with DAPI | Vector Laboratories | H-1200 |
| VECTASTAIN Elite ABC HRP Kit | Vector Laboratories | PK-6100 |
| Wright stain | Sigma-Aldrich | WS16-500ML |
| **Critical commercial assays** | | |
| Click-IT Plus EdU Alexa Fluor 647 Flow Cytometry Assay Kit | Thermo Fisher Scientific | C10634 |
| 10-plex Luminex ProcartaPlex assay | Thermo Fisher Scientific | PPX-10 |
| 6-plex Luminex ProcartaPlex assay | Thermo Fisher Scientific | PPX-06 |
| Magic Red cathepsin assay kit | Bio-Rad | ICT937 |
| **Software and algorithms** | | |
| FlowJo v10.6.1 | Tree Star | https://www.flowjo.com/ |
| R version 3.5.1 | The R Foundation | https://www.r-project.org/ |
| Seurat v3 | Butler et al., 2018 | https://satijalab.org/seurat/ |
| velocyto and velocyto.R | La Manno et al., 2018 | https://github.com/velocyto-team/velocyto.R |
| gProfiler web tool | Reimand et al., 2016 | https://biit.cs.ut.ee/gprofiler/gost |
| Slingshot R package v1.1.3 | Street et al., 2018 | https://github.com/kstreet13/slingshot |
| CellphoneDB | Efremova et al., 2020 | https://www.cellphonedb.org/ |

DAB, 3,3′-diaminobenzidine; MRS, Man, Rogosa, and Sharpe; poly(I:C), polyinosinic:polycytidylic acid.
